# Tailoring the Properties of Ethyl Cellulose Membranes Using Hydrophobic Deep Eutectic Solvents

**DOI:** 10.1021/acssuschemeng.5c13894

**Published:** 2026-04-06

**Authors:** Bhavna Alke, Elena Gabirondo, Vitor D. Alves, João G. Crespo, Carla Brazinha, Liliana C. Tomé

**Affiliations:** † LAQV/REQUIMTE, Department of Chemistry, NOVA School of Science and Technology, NOVA FCT, Universidade NOVA de Lisboa, 2829-516 Caparica, Portugal; ‡ CEMMPRE, ARISE, Department of Chemical Engineering, University of Coimbra, Rua Sílvio Lima, 3030-790 Coimbra, Portugal; § POLYMAT and Department of Polymers and Advanced Materials: Physics, Chemistry and Technology, Faculty of Chemistry, University of the Basque Country UPV/EHU, P° Manuel de Lardizabal, 3, 20018 Donostia-San Sebastian, Spain; ∥ LEAF—Linking Landscape, Environment, Agriculture and Food Research Center, Associate Laboratory TERRA, Instituto Superior de Agronomia, Universidade de Lisboa, 1349-017 Lisboa, Portugal; ⊥ ITQB NOVA, Universidade NOVA de Lisboa, Av. da República, 2780-157 Oeiras, Portugal

**Keywords:** Sustainable membrane fabrication, polymer plasticization, alcohol-based eutectic solvents, gas permeation, water vapor permeability, eco-scale assessment, Hansen solubility interactions

## Abstract

Developing sustainable strategies for the lucrative application of biopolymers is crucial for advancing environmentally friendly materials. Ethyl cellulose, a water-insoluble and biocompatible polysaccharide with excellent mechanical and thermal stability, is a promising material for membrane technology. This study explores nonionic, alcohol-based hydrophobic natural deep eutectic solvents (DES) as green additives for ethyl cellulose membranes. Spectroscopic analyses confirmed the successful incorporation of DES, supported by Hansen solubility parameter predictions indicating dispersive and hydrogen-bonding interactions as key mechanisms. The incorporation of DES markedly enhanced the flexibility of the polymer, lowering the glass transition temperature from 98 °C to below 0 °C. Barrier studies demonstrated that the addition of DES decreased the water vapor permeability and single-gas permeabilities (N_2_, CO_2_, and O_2_) without compromising gas selectivity. Moreover, the Eco-Scale assessment classified the membrane fabrication method as green, confirming improvements in sustainability metrics. Overall, hydrophobic DES are versatile and environmentally benign additives that simultaneously enable the tailoring of mechanical properties and improvement of barrier performance in ethyl cellulose membranes.

## Graphical abstract

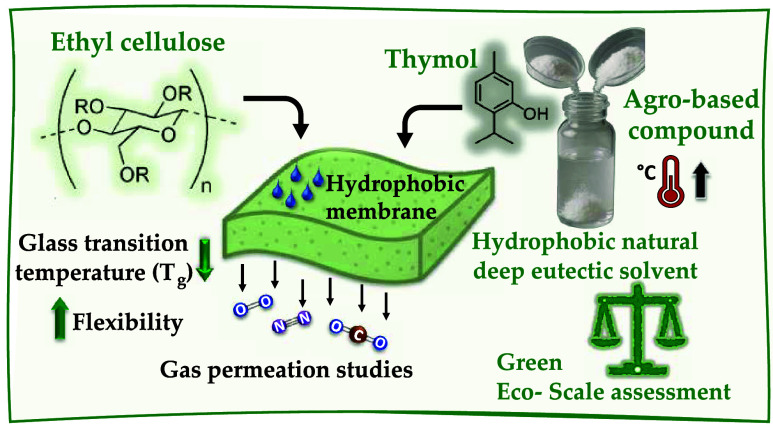

## 1 Introduction

Cellulose is the most abundant naturally occurring polymer.^1^ The abundant availability and favorable properties, including high mechanical strength, stability, nontoxicity, biocompatibility, and biodegradability, make cellulose and its derivatives attractive polymer candidates for membrane production.^2,3^ The applicability of these membranes is largely governed by the nature of the cellulose derivatives, which can be classified as water-soluble or water-insoluble. One notable derivative is ethyl cellulose, a linear water-insoluble polysaccharide that, although not readily biodegradable, has favorable biocompatibility, nontoxicity, and is highly soluble in organic solvents. Moreover, it exhibits advantageous mechanical properties, thermoplastic behavior, and chemical stability compared to its hydrophilic counterparts. These attributes make ethyl cellulose particularly valuable in biotechnology, drug delivery, and membrane-based applications. As a membrane-forming polymer, it offers durability, low cost, and moderate performance in gas separation and pervaporation processes.^4^

Owing to the relatively high glass transition temperature of ethyl cellulose (128–130 °C), the addition of a plasticizer is required to enhance its thermal and mechanical properties. Since the 1970s, numerous studies have investigated the influence of different types and amounts of plasticizers on ethyl cellulose films or coatings, with early applications mainly focused on drug release.^5–9^ Recently, research has expanded to explore broader functionalities. In 2020, Su et al. evaluated castor oil as a plasticizer in capsaicin-containing ethyl cellulose films, highlighting their antimicrobial potential for food packaging applications.^10^ In 2021, ethyl cellulose films prepared using acetic acid as the solvent, combined with lipoic acid and plant oil-derived polyols as plasticizers, demonstrated improved mechanical properties, although at the expense of the thermal stability of ethyl cellulose-based composite films.^11,12^ Shlush et al. employed both solution casting (with acetone) and hot-melt extrusion to produce ethyl cellulose films plasticized with compounds such as glycerol and Myvacet®.^13,14^ In this context, the present study explores the use of hydrophobic natural deep eutectic solvents as additives for ethyl cellulose membranes, offering a novel and sustainable alternative to conventional approaches.

Hydrophobic deep eutectic solvents represent a subcategory of deep eutectic solvents (DES).^15,16^ Their hydrophobic nature renders them immiscible with water, thereby expanding the applicability of DES in nonaqueous environments.^15,17^ Natural hydrophobic DES are typically formed from biodegradable and generally nontoxic compounds, combining a hydrogen bond donor and a hydrogen bond acceptor, with common examples including natural fatty acids, menthol, thymol, and long-chain alcohols.^18–20^ These solvents are characterized by low volatility, tunable physicochemical properties, and excellent solubilizing capabilities for hydrophobic compounds.^18^ In this study, a new class of alcohol-based hydrophobic DES, formed by combining thymol with compounds such as 1-hexadecanol, vanillyl alcohol, and β-sitosterol, was investigated as a novel approach for use as additives.

Plasticizers typically reduce the cohesive intermolecular forces between the polymer chains, increasing the free volume and polymer chain mobility, which in turn enhances the flexibility of the resulting membrane.^21^ The use of DES as plasticizers has been reported in the preparation of biopolymeric membranes.^22,23^ However, most studies have focused on hydrophilic choline chloride (ChCl)-based DES, such as ChCl:urea, ChCl:glycerol, and ChCl:citric acid, used in combination with biopolymers like cellulose, chitosan, starch, and agar.^22,23^ To the best of our knowledge, the use of DES as plasticizers for ethyl cellulose has not yet been reported. Furthermore, the potential of hydrophobic, nonionic DES for membrane plasticization remains unexplored.

Hydrophobic DES are particularly interesting liquid phases for CO_2_ separation membranes.^24^ Their ability to enhance membrane affinity for specific gases and increase gas diffusivity can improve separation performance. Moreover, the composition of hydrophobic DES can be tailored to optimize the separation of target gas pairs, such as CO_2_/CH_4_ or O_2_/N_2_.^25^ In addition to their performance benefits, natural hydrophobic DES are sustainable alternatives to conventional plasticizers because of their low toxicity and biodegradability, making them attractive for eco-friendly gas membrane technologies in industrial, environmental, and energy applications.

In this study, alcohol-based hydrophobic DES were incorporated into ethyl cellulose membranes via solution casting and solvent evaporation. The effects of varying DES concentrations on the thermal and mechanical properties of the membranes were also evaluated. Comprehensive characterization, including spectroscopic and thermal analyses, was conducted to elucidate the possible interactions between the DES and the polymer in the resulting membranes. The barrier properties, such as water vapor and gas permeability, were also assessed to determine the influence of DES as additives on the performance of ethyl cellulose membranes.

## 2 Materials and Methods

### 2.1 Materials

The deep eutectic solvents (DES) were prepared using thymol (≥98.5%), DL-menthol (≥95%), 1-hexadecanol (≥99%), vanillyl alcohol (98%), and β-sitosterol (≥70%), which were purchased from Sigma-Aldrich (USA). The polymer ethyl cellulose (48–49.5% (w/w) ethoxyl basis) was also obtained from Sigma-Aldrich (USA). Ethanol (96%) was acquired from Laborspirit (Portugal).

### 2.2 DES Preparation

Hydrophobic DES were prepared using the conventional heating method in the required molar ratios of the components. The heating was carried out at 80 °C for thymol: menthol (2:1) and thymol: 1-hexadecanol (9:1) and at 100 °C for thymol: vanillyl alcohol (9:1) and thymol: β-sitosterol (9:1) under continuous stirring until a homogeneous liquid mixture was observed. The molar ratios used for the DES formulations were selected based on their stability and reproducible liquid formation. For the thymol: 1-hexadecanol and thymol: vanillyl alcohol systems, our preliminary tests showed that the 9:1 molar ratio resulted in clear and stable liquids over prolonged periods without crystallization or phase separation. In the case of thymol: menthol, the literature reports indicate that molar ratios above 3:1 thymol:menthol (with increasing thymol concentration) tend to become unstable or prone to partial crystallization over time.^26^ To avoid these issues and ensure reproducible membrane casting, we selected a 2:1 thymol: menthol ratio, which consistently formed a stable eutectic mixture under the tested conditions.

The details of the individual compounds are listed in Table 1.

**Table 1:**
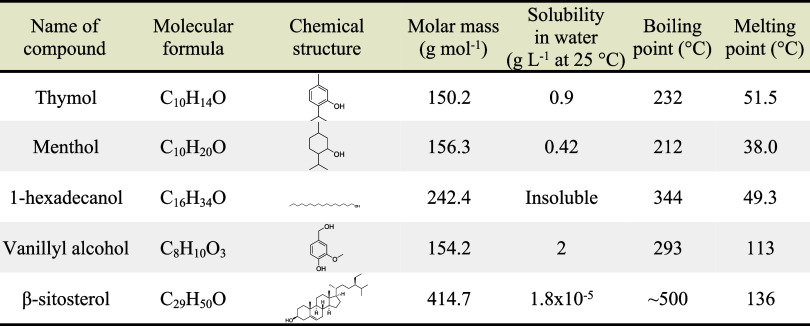
Properties of Compounds Used to Prepare Hydrophobic DES^27^

### 2.3 Membrane Preparation

The membranes were prepared by the solvent casting method using ethanol. Different ratios of ethyl cellulose (polymer) and the desired DES were dissolved in ethanol under continuous stirring at 60 °C. A silicon oil bath was used to maintain a uniform temperature in the glass vial. After complete dissolution, the mixture was poured into a glass Petri dish and placed in an oven maintained at 40 °C to allow the slow evaporation of ethanol. After complete drying, the membranes were peeled off the Petri plates and stored in airtight, zip-locked bags.

### 2.4 Characterization

A Spectrum two FTIR spectrometer (PerkinElmer, USA) was used to record the Fourier transform infrared (ATR-FTIR) spectra of the DES, its individual components, and the membranes, in order to identify possible intermolecular interactions. For each sample, 25 scans were performed from 400 to 4000 cm^–1^ at a resolution of 4 cm^–1^.

^1^H nuclear magnetic resonance (^1^H NMR) spectroscopy of the DES, its individual components, and the membranes was performed using a Bruker Avance DPX 300 MHz spectrometer at 300.16 MHz. Deuterated DMSO was used as the solvent at room temperature for the DES and their individual components, except for those containing β-sitosterol, for which deuterated chloroform was used. Deuterated methanol was used for the membrane samples. The experimental conditions were as follows: 10 mg sample, 3 s acquisition time, 1 s delay time, 8.5 μs pulse, spectral width of 5000 Hz, and 32 scans.

Differential scanning calorimetry (DSC) measurements were carried out using a PerkinElmer 8500 DSC equipped with an Intracooler III. The analysis was performed under a nitrogen atmosphere by crimping samples (∼5 mg) into nonrecyclable aluminum hermetic pans. The samples were initially heated from 25 to 150 °C and maintained isothermally at 150 °C for 3 min to remove their thermal history. The samples were then cooled to −70 °C and maintained isothermally at −70 °C for 10 min. Subsequently, the samples were heated to 150 °C, and the phase transition behavior was analyzed from the second heating cycle. All heating and cooling cycles were performed at a rate of 10 °C/min.

Thermogravimetric analysis (TGA) was performed using a TGA Q500 V20 (TA Instrument). To prevent thermo-oxidative degradation, the samples (∼10 mg) were heated in a nitrogen atmosphere from room temperature to 600 °C at a rate of 10 °C/min. The Universal Analysis software (version 4.5 A) was used to determine the onset (*T*_onset_) and decomposition (*T*_dec_) temperatures.

Hansen solubility parameters (HSP) are widely used to predict the interactions between different substances. In membrane fabrication, HSP have been used to understand polymer solubility by studying the interactions between polymers and solvents.^28,29^ Previous studies have demonstrated that the thermal and mechanical behaviors of ethyl cellulose membranes are strongly influenced by the interactions between the polymer and plasticizer.^13,14^ Therefore, HSP theory has been employed in the current work to study these interactions. According to Hansen solubility parameters (HSP) theory, two components are considered miscible if the difference between their total solubility parameters, δ, is <5 MPa^0.5^.^30^ The total solubility parameter was calculated based on three components: dispersion forces (δ_D_), polarity (δ_P_), and hydrogen bonding (δ_H_), using the following equation

1 $$\delta^{2}=\delta_{D}^{2}+\delta_{P}^{2}+\delta_{H}^{2}$$

The solubility parameter of the mixtures (δ_M_^mix^) were calculated as

2 $$\delta_{M}^{\mathrm{mix}}={\alpha \delta }_{M}^{1}+\left(1-\alpha \right)\delta_{M}^{2}$$

where δ_M_^1^ or δ_M_^2^ could be δ_D,_ δ_P_, or δ_H_ of components 1 or 2 and α was the volume fraction of component 1. Considering hydrogen bond formation, the HSP theory states that closer proximity of the δ_H_ values promotes stronger hydrogen bonding. Based on this, the Hansen solubility parameters were evaluated.

The mechanical properties of the ethyl cellulose membranes were determined using a TA XT Plus Texture analyzer (Stable Micro Systems, Godalming, UK). Each membrane was punctured through a 10 mm diameter hole with a 2 mm diameter probe at a constant velocity of 1 mm s^–1^. The puncture strength (MPa), elongation at break (%), and Young’s modulus (MPa) were determined from the data obtained. All membranes were tested in triplicates.

A ThermoScientific desktop scanning electron microscope (SEM model Phenom ProX G6) was used to obtain surface and cross-sectional images of the ethyl cellulose membranes. The SEM consisted of a CeB6 filament and a light-element energy-dispersive spectroscopy (EDS) detector. The Phenom User Interface was used for digital image acquisition. The samples were initially sputter-coated with a thin Au/Pd film in a Quorum Technologies coater (model Q150T ES) after being placed on an Al stub with double-sided carbon tape.

A Drop Shape Analyzer—DSA25 (KRÜSS, Hamburg, Germany) was used to determine the static water contact angle of the membranes using the sessile drop method, and the results were analyzed using the Advance software. The values reported are the averages of the triplicates.

### 2.5 Eco-Scale Assessment

The reduction of toxic materials, energy expenditure, and waste generation are some of the vital ways in which green and sustainable methodologies can be achieved in membrane research. Many metrics have been established to evaluate the “green” nature of chemical processes. The Eco-Scale assessment, proposed by Gałuszka et al. in 2012, is one of the simplest tools used for green metric analysis.^31^ This semiquantitative tool classifies the greenness level of an experimental method based on the calculation of a numerical score. In this assessment, an ideal green procedure is assigned a total score of 100. Based on the harmful impact of the method on the environment, “penalty” points are subtracted from the score. These penalty points refer to the various negative effects on the environment, including the use of hazardous solvents, high energy consumption, and waste generation. This tool classifies the methods into three categories: green method (score >75), nongreen method (score between 50 and 75), and extremely harmful method to the health/environment (score of <50).^32^ In this study, an Eco-Scale assessment was performed to determine the greenness of the additives using the methodology detailed by Gałuszka et al. and van Aken et al.^31,33^

### 2.6 Water Vapor Permeability

The water vapor permeabilities of the ethyl cellulose membranes were measured using a gravimetric method. The experimental setup is described elsewhere.^34^ A volume of 9 mL of saturated sodium chloride solution (*a*_w_ = 0.755) was transferred to cylindrical glass cups, and the cups were sealed with the sample (3 cm diameter of film was exposed) using an aluminum tape. The glass cups were placed inside a desiccator containing a saturated solution of magnesium chloride (*a*_w_ = 0.328). The resistance to mass transfer above the membrane was reduced by placing a fan inside the desiccator. A digital thermohygrometer (TFA Dostmann/Kat. Nr. 30.5013, Germany) was used to monitor the relative humidity and temperature inside the desiccator. The glass cups were weighed before the experiment and at regular intervals for 8 h. All experiments were performed in triplicates. The water vapor permeability (WVP) was determined as follows

3 $$\mathrm{WVP}=\frac{N_{w}\times \delta }{{\Delta p}_{w,\mathrm{eff}}}$$

where *N*_w_ (mol m^–2^ s^–1^) is the water vapor flux, δ (m) is the film thickness, and Δ*p*_w,eff_ (Pa) is the effective driving force, expressed as the water vapor pressure difference between both sides of the film.

### 2.7 Gas Permeation

The single gas permeabilities (N_2_, CO_2_, and O_2_) of the ethyl cellulose membranes were measured in triplicates using a time-lag setup, as illustrated in Figure 1. The setup consisted of a feed compartment connected to a gas reservoir, where a backpressure controller was used to maintain a constant feed pressure. A membrane module separates the feed and permeate compartments. A vacuum pump was connected to the permeate compartment of the membrane module.

**Figure 1:**
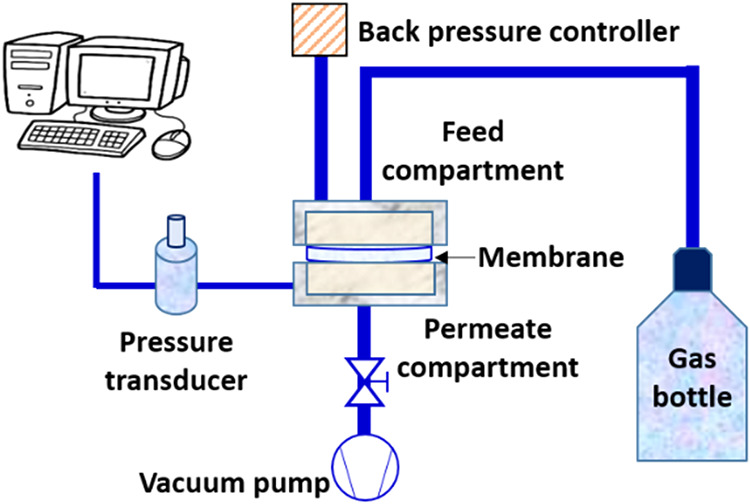
Schematic illustration of the time-lag setup.^34^

Initially, once the membrane was placed in the module, vacuum was supplied to the permeate compartment to remove any trapped gases and ensure complete degassing of the membrane. The vacuum valve was then closed, and the feed gas was introduced at a constant feed pressure of 1.05 bar. The pressure variations in the permeate compartment were continuously recorded in real time using a transducer connected to the compartment.

The transport was defined using (eq 4), where *J*_mol_ (mol m^–2^ s^–1^) is the molar flux (the ratio between the molar flow rate *Q*_mol_ (mol s^–1^) and the membrane area *A* (m^2^)), *P*_mol_ (mol m^–1^ s^–1^ Pa^–1^) is the molar permeability, Δ*p* (Pa) is the driving force calculated by the difference between the feed and permeate pressures, and δ (m) is the membrane thickness

4 $$J_{\mathrm{mol}}=\frac{P_{\mathrm{mol}}\times \Delta p}{\delta }$$

The volumetric gas flow rate *Q*_vol_ (Pa m^3^ s^–1^) and the corresponding molar flow rate *Q*_mol_ (mol s^–1^) are given by the following equations

5 $$Q_{\mathrm{vol}}=\frac{dp}{dt}\times V_{\mathrm{perm}}$$

6 $$Q_{\mathrm{mol}}=\frac{1}{\mathrm{RT}}\times Q_{\mathrm{vol}}$$

where *T* is the operating temperature (K). Subsequently, the permeability *P* was calculated using (eq 7)­

7 $$\mathrm{permeability}\;\left(P\right)=\frac{Q_{\mathrm{mol}}\times \delta }{\Delta p\times A}$$

The solubility was calculated by the relation

8 $$S=\frac{P}{D}$$

where *S* (cm^3^ (STP) cm^–3^ MPa^–1^) is the solubility and *D* (cm^2^ s^–1^) is the diffusivity given by

9 $$D=\frac{\delta^{2}}{6\theta }$$

where δ (cm) is the thickness of the membrane and θ (s) is the time-lag.

The ideal gas selectivity of gas pairs A and B was calculated by (eq 10), where *P*_A_ and *P*_B_ are the permeabilities of gas A and B respectively.

10 $$\mathrm{selectivity}\;\left(A/B\right)=\frac{P_{A}}{P_{B}}$$

## 3 Results and Discussion

The DES used in this study, namely thymol: menthol (2:1), thymol: 1-hexadecanol (9:1), thymol: vanillyl alcohol (9:1), and thymol: β-sitosterol (9:1), were prepared using a heating method. The FTIR spectra of the prepared DES and their starting materials are shown in Figure S1 (Supporting Information). The broad band between 3650 and 3250 cm^–1^ indicates the formation of hydrogen bonds, which are characteristic of DES. In addition, the disappearance or shift of the −OH signals observed in the ^1^H NMR spectra (Figure S2) further confirmed the successful preparation of DES. Figures S3–S6 depict the ^1^H NMR spectra of the different DES used in the current study. The prepared DES were stored overnight at 30 °C to ensure stability.

Ethyl cellulose membranes were then prepared by solvent casting, as described previously by incorporating 0, 20, 50, and 80 wt % DES into the polymer matrix. When selecting an effective solvent to dissolve a specific polymer, the polarity and dielectric constant of the solvent must be considered. The most commonly used solvents for ethyl cellulose are acetone, ethanol, and ethyl acetate. Acetone and ethanol are polar solvents with high dielectric constants, ethyl acetate has a lower dielectric constant and is less polar.^14^ Thus, acetone and ethanol are considered more effective solvents for ethyl cellulose. However, because acetone has a weaker hydrogen bonding capacity, ethanol is considered more effective for the dissolution of ethyl cellulose.^35^ Therefore, in the current study, ethanol was used as the solvent for ethyl cellulose polymer.

The pictures of ethyl cellulose membranes containing different DES is shown in Figures 2 and S7. When thymol: 1-hexadecanol (9:1) or thymol: β-sitosterol (9:1) were used, no homogeneous membranes were obtained, as the DES precipitated during solvent evaporation (Figure S7). In contrast, membranes prepared with thymol: menthol (2:1) were successfully formed at all tested DES concentrations. However, for thymol: vanillyl alcohol (9:1), no homogeneous membranes were obtained when the DES content exceeded 50 wt % (Figure S7). These results demonstrate that membrane formation depends not only on the type of DES employed but also on its concentration.

**Figure 2:**
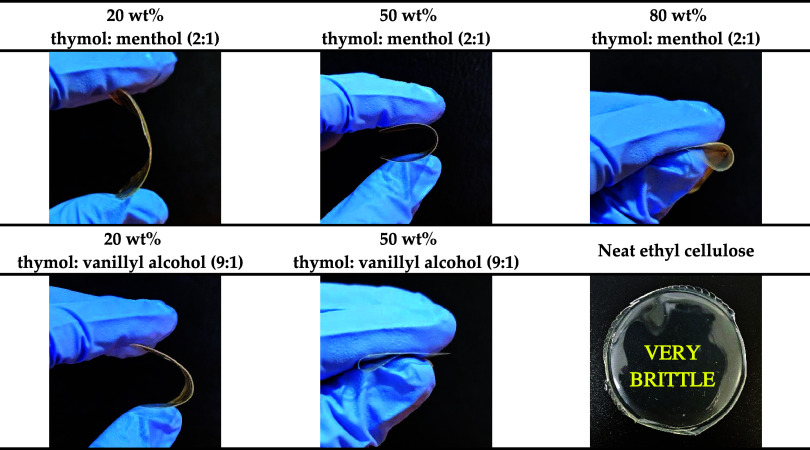
Pictures of the ethyl cellulose membranes successfully prepared.

### 3.1 Membrane Characterization

Spectroscopic studies were conducted to confirm the presence of DES in the prepared membranes. The ^1^H NMR spectra (Figure S8) clearly show DES specific peaks. Multiple peaks were observed in the membranes with DES, which were absent in ethyl cellulose but present in DES.

The FTIR spectra of the membranes containing different percentages of DES were compared with those of neat ethyl cellulose to assess the possible molecular interactions between DES and the polymer matrix (Figure 3).

**Figure 3:**
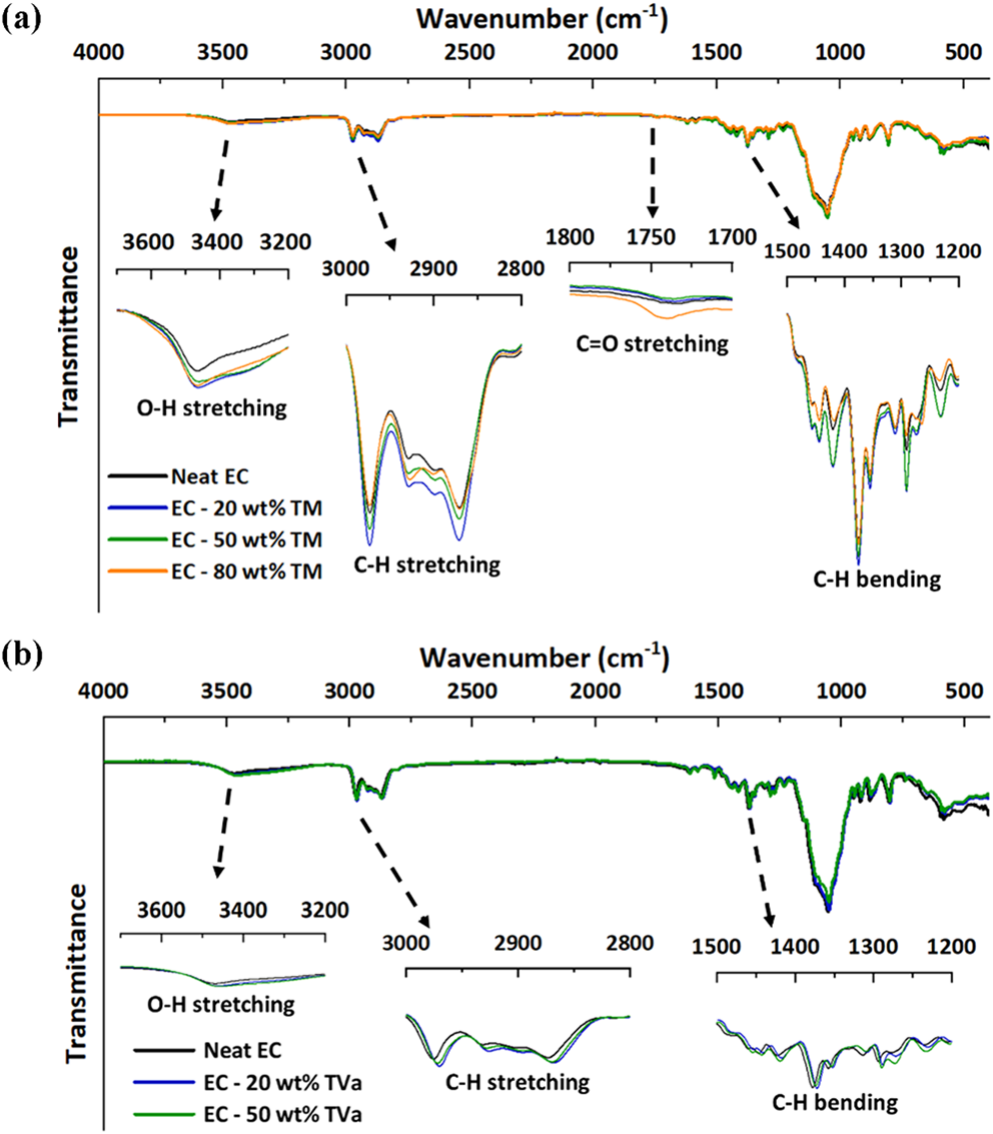
FTIR spectra of ethyl cellulose (EC) membranes combining different amounts of DES: (a) TM = thymol: menthol (2:1) and (b) TVa = thymol: vanillyl alcohol (9:1).

All samples displayed a distinct absorption band near 1050 cm^–1^, characteristic of the C–O–C stretching vibrations of ethyl cellulose. A broad absorption in the 3700–3200 cm^–1^ range was also observed, which was associated with the O–H stretching. The bands in the 3000–2800 cm^–1^ and 1500–1200 cm^–1^ ranges are assigned to the C–H stretching and bending modes of the methyl and methylene groups.

Although the overall spectral profiles were consistent across the samples, gradual changes were observed with increasing DES content. Notably, the band around 1745 cm^–1^ showed variations in intensity upon the addition of thymol: menthol (2:1). This band, typically assigned to C=O stretching vibrations, suggests the presence of specific interactions, likely hydrogen bonding or dipole–dipole forces, between the functional groups of the thymol: menthol (2:1) DES and the ethyl cellulose chains. These effects were more pronounced in the membrane containing 80 wt % thymol: menthol (2:1), indicating stronger molecular-level interactions at higher DES concentrations. Additionally, the broadening of the O–H stretching region (∼3500 cm^–1^) was observed upon DES addition, indicating the involvement of hydroxyl groups from both ethyl cellulose and DES in hydrogen bonding interactions. Changes in the alkyl C–H stretching region (3000–2800 cm^–1^) were also observed, suggesting enhanced van der Waals interactions between the polymer and hydrophobic components of the DES. Overall, these results demonstrate that DES interact with ethyl cellulose through hydrogen bonding and dispersive forces. DSC and TGA analyses were carried out to evaluate the thermal properties of the prepared ethyl cellulose membranes containing DES. As shown in Figure 4 and Table 2, these properties were compared with those of neat ethyl cellulose.

**Figure 4:**
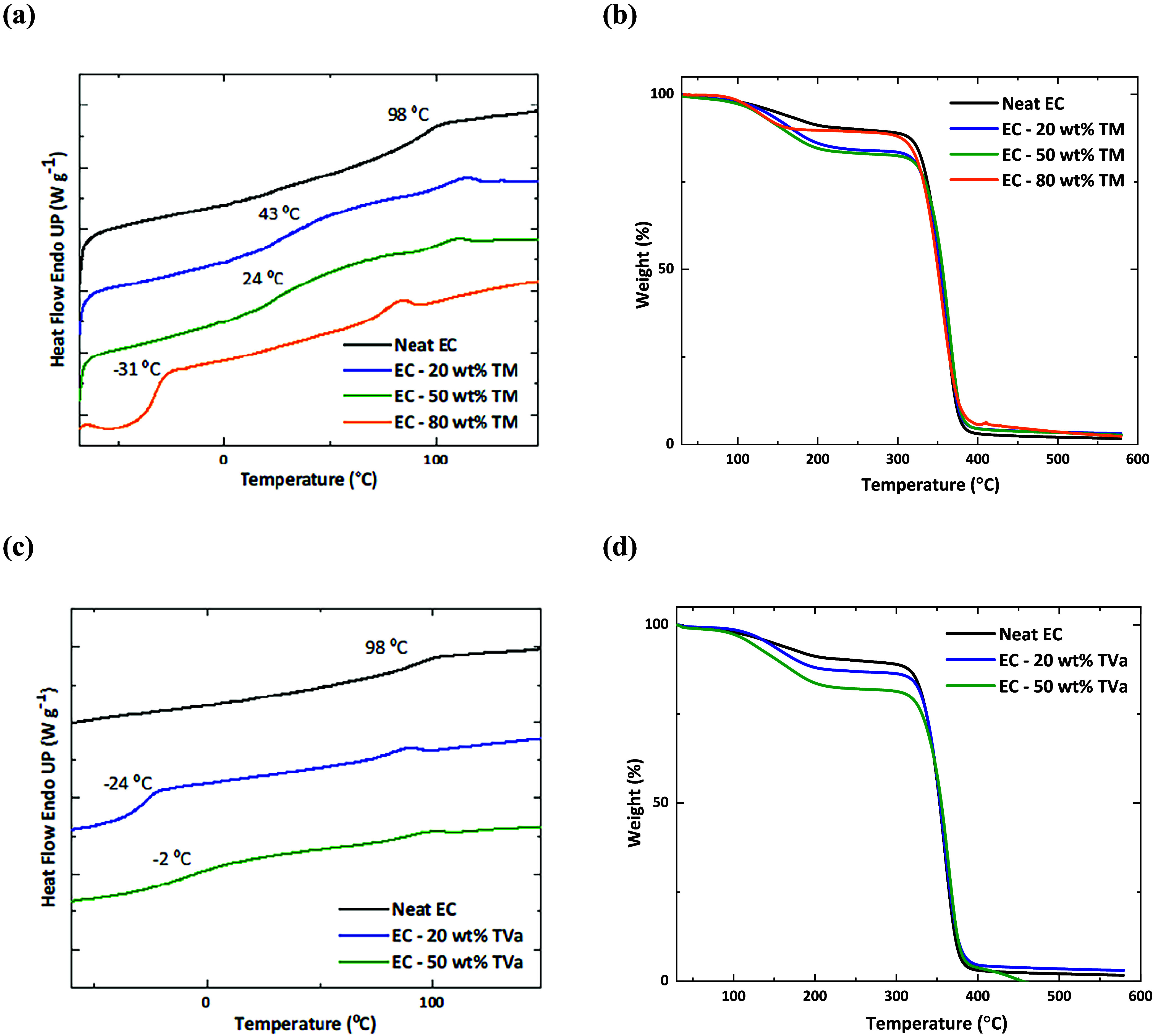
DSC and TGA analyses of the ethyl cellulose (EC) membranes with different amounts of DES: (a) DSC and (b) TGA of membranes containing TM = thymol: menthol (2:1); (c) DSC and (d) TGA of membranes containing TVa = thymol: vanillyl alcohol (9:1).

**Table 2:** Evaluation of the Thermal Properties of Ethyl Cellulose (EC) Membranes with Different Amounts of DES: TM = thymol: menthol (2:1) and TVa = thymol: vanillyl alcohol (9:1)

membrane	*T*_onset_ ^a^ (°C)	*T*_dec_ ^b^ (°C)	*T*_g_ ^c^ (°C)
Neat ethyl cellulose	320	353	98
EC-20 wt % TM	323	353	43
EC-50 wt % TM	324	356	24
EC-80 wt % TM	320	353	–31
EC-20 wt % TVa	325	354	–24
EC-50 wt % TVa	324	355	–2

a *T*_onset_ (onset temperature) is the temperature at which the baseline slope changes during heating.

b *T*_dec_ (decomposition temperature) is the temperature at which 50% weight loss occurs.

c *T*_g_ (glass transition temperature) is the temperature at the midpoint of the glass transition region.

The glass transition temperature (*T*_g_) values clearly demonstrated that the incorporation of DES led to a significant reduction in *T*_g_, depending on the type and amount of DES incorporated in the membrane. Notably, only 20 wt % thymol: vanillyl alcohol (9:1) was sufficient to produce a membrane with a negative *T*_g_ (−24 °C), whereas 80 wt % thymol: menthol (2:1) was required to achieve a similar effect (−31 °C). On the other hand, the TGA results demonstrated that the addition of DES did not significantly impact the thermal stability of the ethyl cellulose membranes. Regardless of the DES type or content (even up to 80 wt %), the decomposition temperature corresponding to 50% mass loss remained close to 350 °C.

In this study, the interactions among ethyl cellulose, ethanol, and DES (used as plasticizers) were evaluated using HSP theory, as summarized in Table 3. Both conditions, before and after ethanol evaporation, were considered. According to the HSP theory, two substances are considered miscible when the difference in their total solubility parameters is *<* 5 MPa^0.5^.^30^ As shown in Table 3, the total solubility parameters of the DES are much closer to those of ethyl cellulose than to those of ethanol. This indicates that the DES promoted polymer solubilization, as observed during the experiments.

**Table 3:** Hansen Solubility Parameters (HSP) of Ethyl Cellulose, Ethanol, and the Different DES

		HSP (MPa^0.5^)				
S. No.	component	dispersion (δ_D_)	polarity (δ_P_)	H-bonding (δ_H_)	total (δ)	refs
1	Ethyl cellulose	19.0	5.6	4.9	20.4	^36^
2	Ethanol	15.8	8.8	19.4	26.5	^37^
3	Thymol: menthol (2:1)	18.1	4.6	10.7	21.6	This work
4	Thymol: vanillyl alcohol (9:1)	19.0	4.9	10.8	22.5	
5	Thymol: 1-hexadecanol (9:1)	18.9	5.1	10.1	22.0	
6	Thymol: β-sitosterol (9:1)	19.0	4.8	10.7	22.3	

The HSP theory also states that hydrogen bonding is favored when the δ_H_ values of two substances are in close proximity. As shown in Table 3, the δ_H_ values of the DES (5.2–5.9 MPa^0.5^) are much closer to that of ethyl cellulose (4.9 MPa^0.5^) than to that of ethanol (19.4 MPa^0.5^), indicating that hydrogen bond formation between the DES and the polymer is more favorable than that between ethanol and the polymer. Consequently, the DES can still interact with ethyl cellulose in the presence of ethanol. After ethanol evaporation, these hydrogen bond interactions persisted, enhancing membrane flexibility. In addition, the presence of DES did not significantly affect the overall thermal properties of the membranes (Figure 4), except for *T*_g_, which showed a pronounced decrease as the DES content increased. This reduction can be attributed to the favorable DES–polymer interactions, resulting in increased chain mobility and improved membrane flexibility.

The mechanical properties of the prepared membranes were evaluated using puncture tests, and the obtained stress–strain curves are shown in Figure S9. From the obtained data, Young’s modulus (MPa), fracture strain (%), and tensile strength (MPa) were calculated (Figure 5), which showed that the incorporation of DES had pronounced effect on the mechanical behavior of membranes.

**Figure 5:**
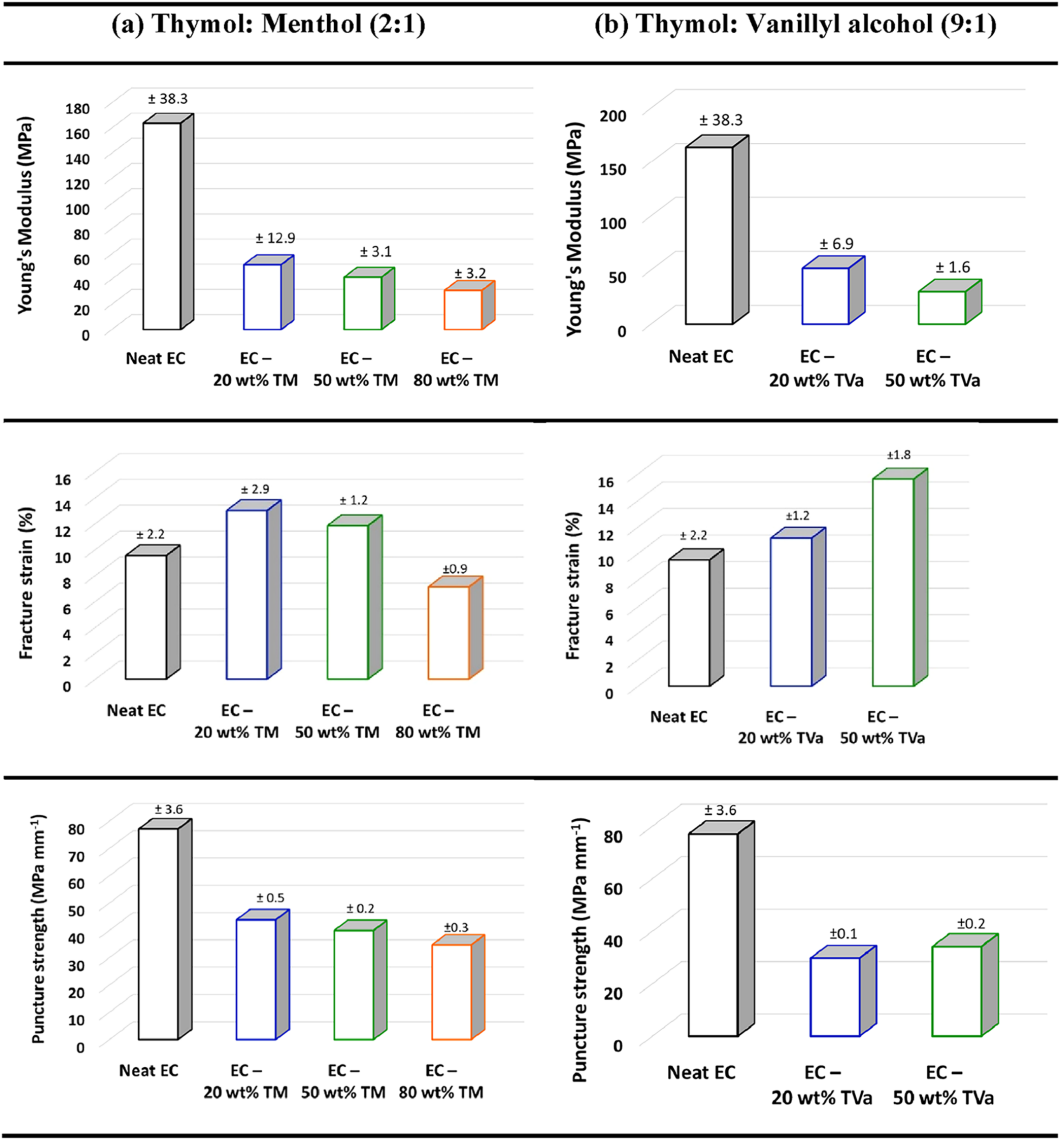
Mechanical properties of the ethyl cellulose (EC) membranes containing different amounts of DES: (a) TM = thymol: menthol (2:1) and (b) TVa = thymol: vanillyl alcohol (9:1). Note: Puncture strength was normalized dividing by membrane thickness (mm).

Young’s modulus decreased significantly with increasing DES content. This is attributed to the plasticizing effect of the DES, which weakens the intermolecular interactions between ethyl cellulose chains, loosening the internal structure of the membrane. This is consistent with the fracture strain results, which showed that membrane ductility increased with DES incorporation and was strongly dependent on the DES content (Figure 5). Conversely, the puncture strength decreased with the addition of both DES types. At around 50 wt % DES, the membranes withstood substantial deformation, while higher DES contents led to a decrease in the fracture strain below that of neat ethyl cellulose. This finding indicates that excessive DES content compromises membrane integrity.

Moreover, the nonlinear behavior observed in the fracture strain of the thymol–menthol containing membranes at concentrations of 50% and above is likely related to changes in the membrane morphology. As shown in the SEM images (Figure 6), these compositions exhibit evidence of phase separation, which can lead to the formation of microvoids within the polymer matrix. The presence of such voids reduces structural uniformity and weakens the load-bearing capability of the membrane, resulting in a decrease in fracture strain. Additionally, excessive plasticizer content at higher DES concentrations may disrupt polymer–polymer entanglements, further diminishing mechanical integrity and contributing to the observed decline in fracture strain. Overall, the mechanical studies clearly demonstrated that the incorporation of DES plays a key role in membrane flexibility, which can be tailored by changing the chemical nature and content of the DES.

**Figure 6:**
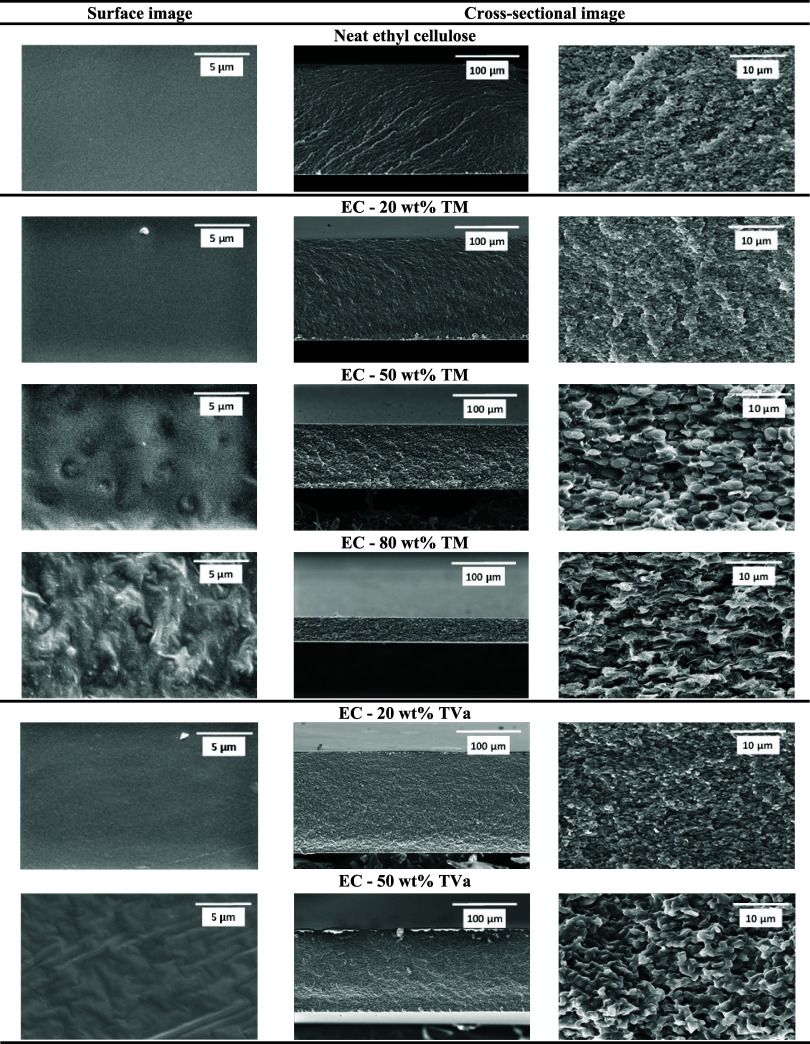
Surface and cross-sectional SEM images of ethyl cellulose (EC) membranes containing different amounts of DES/TM = thymol: menthol (2:1) and TVa = thymol: vanillyl alcohol (9:1).

SEM analysis was performed to investigate the morphological changes in the membranes upon the incorporation of different DES. As shown in the surface images (Figure 6), all membranes exhibited a nonporous structure, regardless of the DES type or content. However, increasing the DES content appeared to enhance the surface roughness, consistent with the increase in the contact angle (Figure 7), which reflects the higher hydrophobicity of the DES.

**Figure 7:**
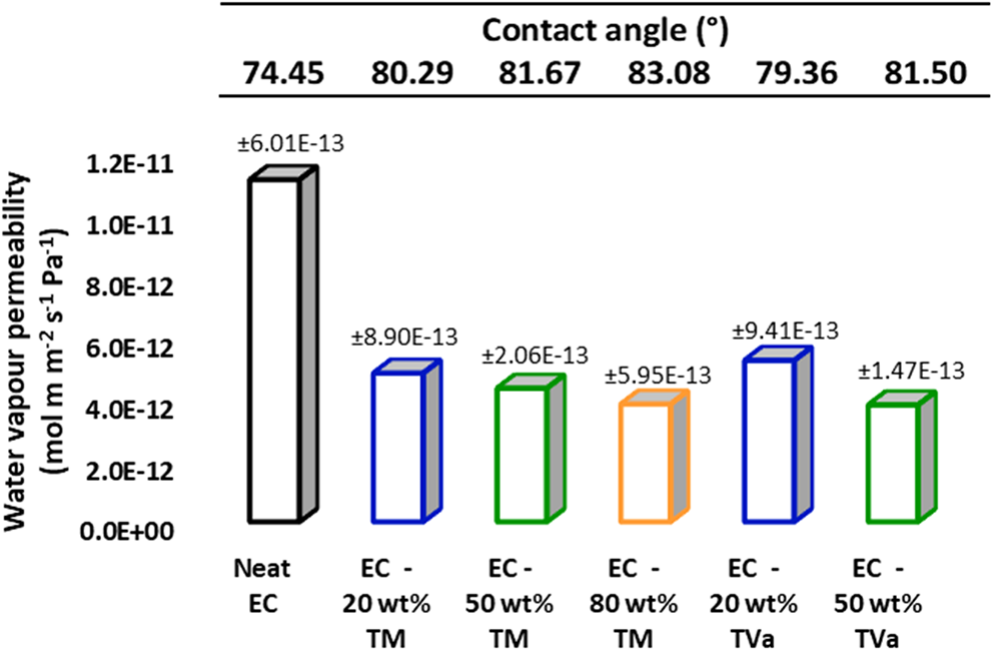
Water vapor permeability and contact angle of ethyl cellulose membranes with different amounts of DES/TM = thymol: menthol (2:1) and TVa = thymol: vanillyl alcohol (9:1).

Cross-sectional images revealed distinct morphological changes. As the amount of DES increased, the membrane structure transitioned from a compact and densely packed arrangement to a more loosely packed morphology. Moreover, at DES contents above 50 wt %, the morphology suggests the onset of phase separation, although this was not visible to the naked eye.

This effect is probably associated with the weakening of intermolecular interactions between ethyl cellulose chains, while the additional DES further enhances its plasticizing effect. Representative SEM images at different magnifications are provided in Figures S10 and S11 in the Supporting Information.

### 3.2 Water Vapor Permeability

Prior to the water vapor permeability experiments, all membranes were prepared, dried in an oven at 35 °C, and stored in airtight bags for further analysis. Figure 7 shows the water vapor permeability values of the membranes containing different types and amounts of DES. The neat ethyl cellulose membrane exhibited a water vapor permeability of (1.11 ± 0.06) × 10^–11^ (mol m m^–2^ s^–1^ Pa^–1^). The incorporation of 20 wt % DES reduced the water vapor permeability by more than half compared to that of the neat ethyl cellulose membrane. Slight additional decreases were observed with an increase in the amount of DES incorporated. It seems that a concentration of 20% imparts the polymeric matrix with sufficient hydrophobicity to hinder water vapor transfer. Contact angle measurements further demonstrated that a higher DES content led to an increase in the surface hydrophobicity of the membranes. A clear relation between the water contact angle and water vapor permeability is observed in Figure 7, indicating that DES incorporation not only increases the hydrophobic nature but also effectively reduces the transport of water vapor through ethyl cellulose membranes.

Furthermore, the membranes do not exhibit hygroscopic behavior. To verify this, water uptake tests were performed by immersing the membranes in water for 8 days (Figure S12). No significant water uptake or dimensional changes were observed, indicating that water absorption under ambient conditions is negligible.

### 3.3 Gas Permeation

Single gas N_2_, CO_2_, and O_2_ permeation experiments were conducted using the time-lag method. The obtained permeability and ideal selectivity values are listed in Table 4.

**Table 4:** Single Gas Permeability (*P*) and Ideal Selectivity (α) Values of Ethyl Cellulose (EC) Membranes with Different Amounts of DES: TM = thymol: menthol (2:1) and TVa = thymol: vanillyl alcohol (9:1)

	Neat EC	EC–20 wt % TM	EC–50 wt % TM	EC–80 wt % TM	EC–20 wt % TVa	EC–50% TVa
*P* (Barrer)						
N_2_	3.78 ± 0.1	3.14 ± 0.04	2.31 ± 0.03	2.69 ± 0.3	2.61 ± 0.3	2.01 ± 0.3
CO_2_	70.8 ± 1.0	64.3 ± 1.3	50.3 ± 1.9	65.4 ± 6.1	57.8 ± 0.9	41.6 ± 3.0
O_2_	20.7 ± 0.6	14.3 ± 1.8	12.6 ± 0.4	10.1 ± 0.9	10.7 ± 1.2	8.13 ± 0.4
Ideal Selectivity (α)						
CO_2_/N_2_	18.7	20.5	21.8	24.4	22.2	20.7
O_2_/N_2_	5.5	4.6	5.5	3.8	4.1	4.0

Figure 8 shows the gas permeability, diffusivity, and solubility of ethyl cellulose membranes. Overall, incorporating DES into the ethyl cellulose membranes led to a decrease in CO_2_ and O_2_ permeability as the DES content increased, suggesting an enhancement in the gas barrier performance. The only exception was the membrane containing 80 wt % thymol: menthol (2:1) DES, which exhibited a slight increase in CO_2_ and N_2_ permeability, which can be attributed to the formation of more flexible domains, microcavities, or interfaces with higher segmental mobility resulting from phase separation, as observed in the SEM images (Figure 6). In contrast, the decrease in O_2_ permeability with increasing DES concentration may be partially related to the antioxidant properties of the DES components.

**Figure 8:**
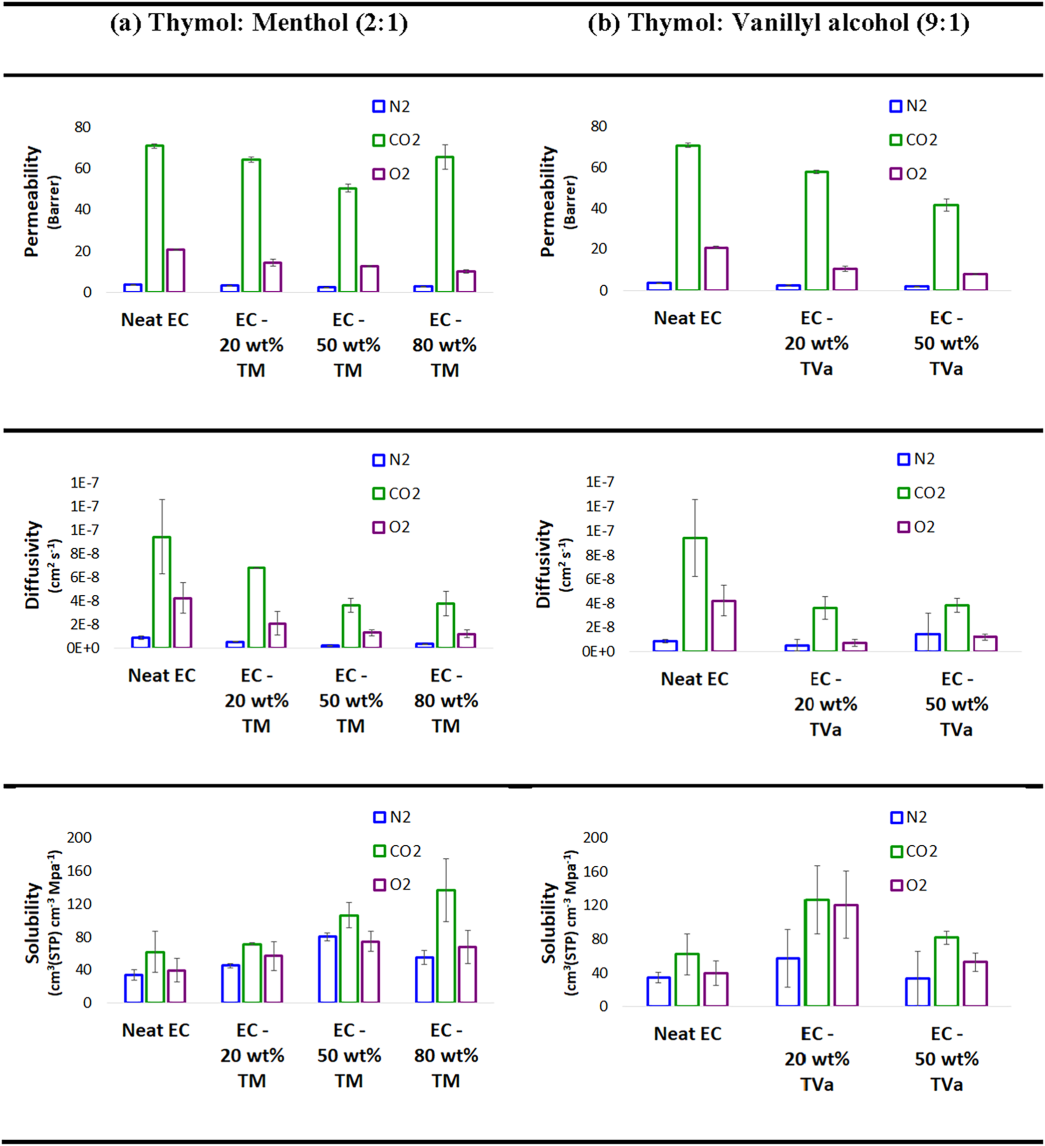
Gas permeation analysis of ethyl cellulose (EC) membranes with different amounts of DES: (a) TM = thymol: menthol (2:1) and (b) TVa = thymol: vanillyl alcohol (9:1).

In addition, with an increase in the DES content, the solubility of CO_2_ increased, whereas the diffusivity decreased (Figure 8). This can be attributed to the increased polarity of the membrane containing DES, which offers more favorable sorption sites and thus enhances solubility. CO_2_ being a quadrupole molecule, interacts preferentially with polar or polarizable groups in a material. The DES components (thymol, menthol, and vanillyl alcohol) contain −OH groups, which enhance the polarity of the membrane. Consequently, comparing membranes with 20 wt % thymol: menthol and thymol: vanillyl alcohol, the CO_2_ solubility in the latter was nearly twice that of the former. This highlights that increasing the polarity of the membrane enhances the CO_2_ solubility. However, this effect appears to be counterbalanced by the lower diffusivity observed, explaining why the overall CO_2_ permeability does not increase as might have been expected.

Finally, the incorporation of DES did not significantly affect the ideal gas selectivity (Table 4). The obtained values are consistent with those reported in the literature for nonfunctionalized ethyl cellulose membranes prepared by solvent casting.^4,38–40^ These results indicate that the incorporation of DES does not compromise the gas selectivity of ethyl cellulose membranes.

### 3.4 Eco-Scale Assessment

Ethyl cellulose membranes have been reported previously using the solvent casting method.^9–11,41,42^ These studies included variations in both the type of plasticizer and the solvent employed. An eco-scale assessment for these reported studies along with the current study was performed. The details of this assessment have been included in Section S3 of the Supporting Information. For all cases, a yield of 100% and the use of low-cost reagents were assumed. The corresponding eco-scale scores were compared with those obtained in this study.

As shown in Table 5, the greenness of a method is largely determined by the reagents used, particularly the solvent choice. Among the three solvents compared, ethanol (penalty points = 5) was the most sustainable option, whereas chloroform (penalty points = 20) was highly toxic. To further illustrate this point, we compared studies in which Dibutyl sebacate (DBS) was used as a common plasticizer. When DBS was combined with ethanol, the process achieved an eco-scale score of 91.^9^ In contrast, a later study using chloroform as the solvent and Vitamin D3 and Vitamin E as additional plasticizers showed a markedly reduced eco-scale score, highlighting the negative impact of introducing toxic solvents and additional reagents.^42^

**Table 5:**
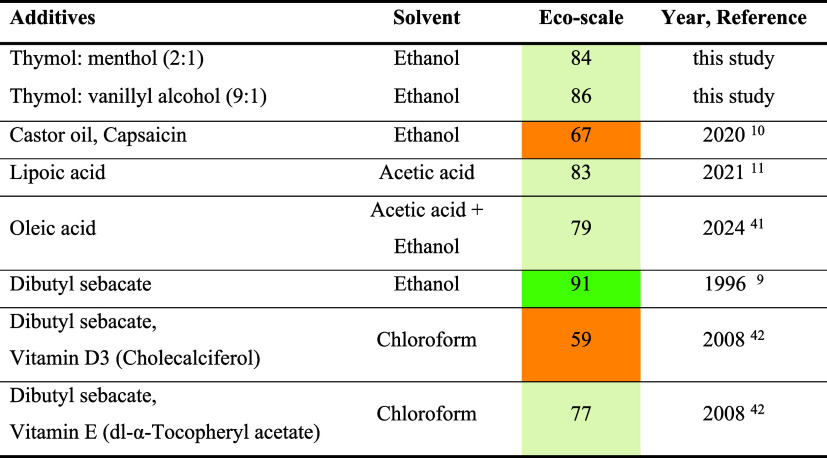
Eco-Scale Assessment of Selected Works on the Preparation of Ethyl Cellulose Membranes Using the Solvent Casting Method

In the present study, highly favorable eco-scale scores were obtained (84 with thymol: menthol (2:1) and 86 with thymol: vanillyl alcohol (9:1)). These results demonstrate that incorporating natural hydrophobic DES improves the plasticization effect and, consequently, the mechanical properties of the membranes, enhancing the overall sustainability of the process. When establishing new methodologies for membrane preparation, it is crucial to evaluate not only the performance of the membranes but also the overall greenness of the preparation system.

## 4 Conclusion

This study proposes the use of nonionic, alcohol-based hydrophobic DES as effective additives for preparing ethyl cellulose membranes via the solvent casting method. Spectroscopic analysis and Hansen solubility parameters indicated polymer-DES interactions, mainly through dispersive forces and hydrogen bonding.

The thermal and mechanical characterization showed that the incorporation of DES within ethyl cellulose chains significantly enhanced membrane flexibility, reducing the glass transition temperature (*T*_g_) from 98 °C to below 0 °C. These findings support the plasticizer effect of DES in the polymeric membrane. The SEM images showed changes in the membrane morphology, which adopted a spongier structure in the presence of the DES. Furthermore, replacing DL-menthol with vanillyl alcohol as the hydrogen bond donor allowed similar mechanical properties to be achieved at a lower DES content. The presence of DES reduced the water vapor permeability by more than half and decreased the gas permeabilities (N_2_, CO_2_, and O_2_) without compromising the gas selectivity (CO_2_/N_2_, O_2_/N_2_).

Overall, these findings highlight the benefits of DES in tailoring the mechanical properties and improving the thermal properties while maintaining the barrier performance or enhancing it in the case of O_2_. An eco-scale assessment confirmed the sustainability of the preparation method, categorizing the use of hydrophobic DES as a green approach. Future studies could explore the modification of DES components to tailor specific membrane properties for targeted applications, such as improved gas solubility, pervaporation, selective separation of organic compounds, increased surface hydrophobicity, and antimicrobial activity.

## Supplementary Material



## Notes

Additional information on Fourier Transport Infrared Spectroscopy and Proton Nuclear Magnetic Resonance results of the DES. Mechanical studies (Stress–Strain curve), Scanning Electron Microscopy images, and Water Uptake of the membranes. Eco-Scale assessment calculations (PDF)

## ○

Department of Bioengineering and iBB - Institute for Bioengineering and Biosciences at Instituto Superior Técnico, Universidade de Lisboa, Lisboa, Portugal
