# Supplementary material for: Tailoring the Properties of Ethyl Cellulose Membranes Using Hydrophobic Deep Eutectic Solvents
Source: ACS Sustain Chem Eng. 2026 Apr 6;14(15):7262–74. doi: 10.1021/acssuschemeng.5c13894 (PMC13103937; doi:10.1021/acssuschemeng.5c13894)
Supplement: Supplementary file 1 [file sc5c13894_si_001.pdf]

SUPPORTING INFORMATION

Tailoring the Properties of Ethyl Cellulose Membranes Using Hydrophobic Deep Eutectic Solvents

Bhavna Alke[†,#], *Elena Gabirondo* [†,‡,§,#], *Vitor D. Alves*[‖],

João G. Crespo[†,⊥], *Carla Brazinha*[*,†], *Liliana C. Tomé*[*,†,‡]

[†]LAQV/REQUIMTE, Department of Chemistry, NOVA School of Science and Technology, NOVA FCT, Universidade NOVA de Lisboa, 2829-516, Caparica, Portugal

[‡]CEMMPRE, ARISE, Department of Chemical Engineering, University of Coimbra, Rua Sílvio Lima, 3030-790, Coimbra, Portugal

[§]POLYMAT and Department of Polymers and Advanced Materials: Physics, Chemistry and Technology, Faculty of Chemistry, University of the Basque Country UPV/EHU, P° Manuel de Lardizabal, 3, 20018 Donostia-San Sebastian, Spain

[‖]LEAF-Linking Landscape, Environment, Agriculture and Food Research Center, Associate Laboratory TERRA, Instituto Superior de Agronomia, Universidade de Lisboa, 1349-017, Lisboa, Portugal

[⊥]ITQB NOVA, Universidade NOVA de Lisboa, Av. da República, 2780-157, Oeiras, Portugal

[#]These authors contributed equally to this work

*Email: Liliana C. Tomé (liliana@eq.uc.pt)

Carla Brazinha (carla.brazinha@tecnico.ulisboa.pt)

Supporting information contains the following:

Number of pages: 16

Number of figures: 12

Number of tables: 1

S1. Solvent characterization

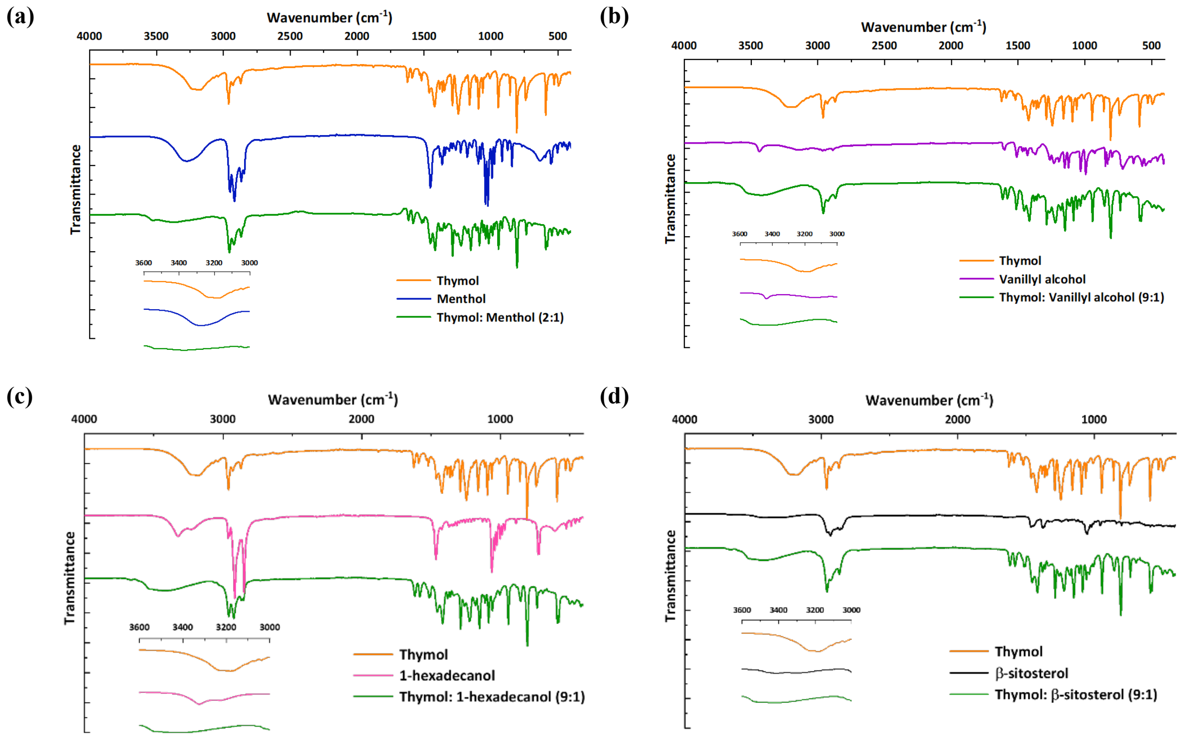

Figure S1. FTIR spectra of the DES and their starting components: **(a)** Thymol: Menthol (2:1) **(b)** Thymol: 1-hexadecanol (9:1) **(c)** Thymol: Vanillyl alcohol (9:1) and **(d)** Thymol: β-sitosterol (9:1).

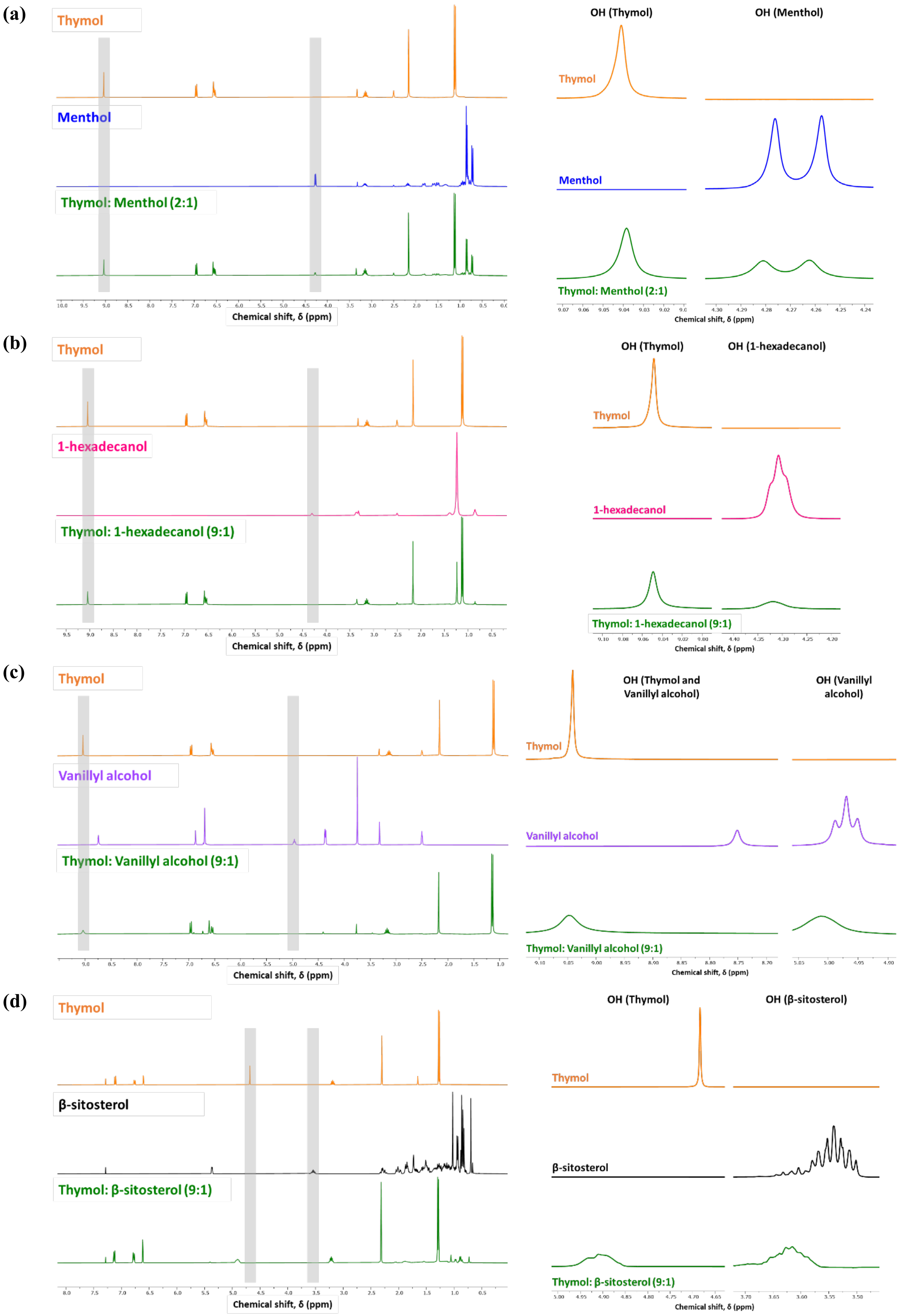

Figure S2. ^{1}H-NMR spectra of DES and their starting components **(a)** Thymol: Menthol (2:1) **(b)** Thymol: 1-hexadecanol (9:1), **(c)** Thymol: Vanillyl alcohol (9:1) and **(d)** Thymol: β-sitosterol (9:1).

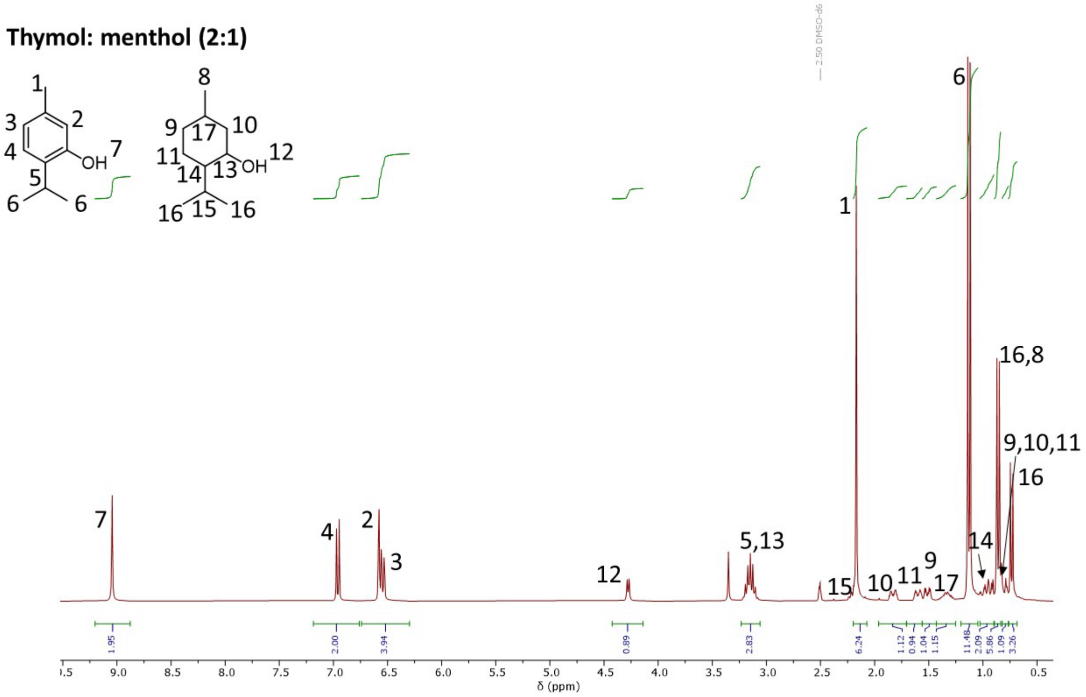

Figure S3. ^{1}H-NMR spectra of DES thymol: menthol (2:1).

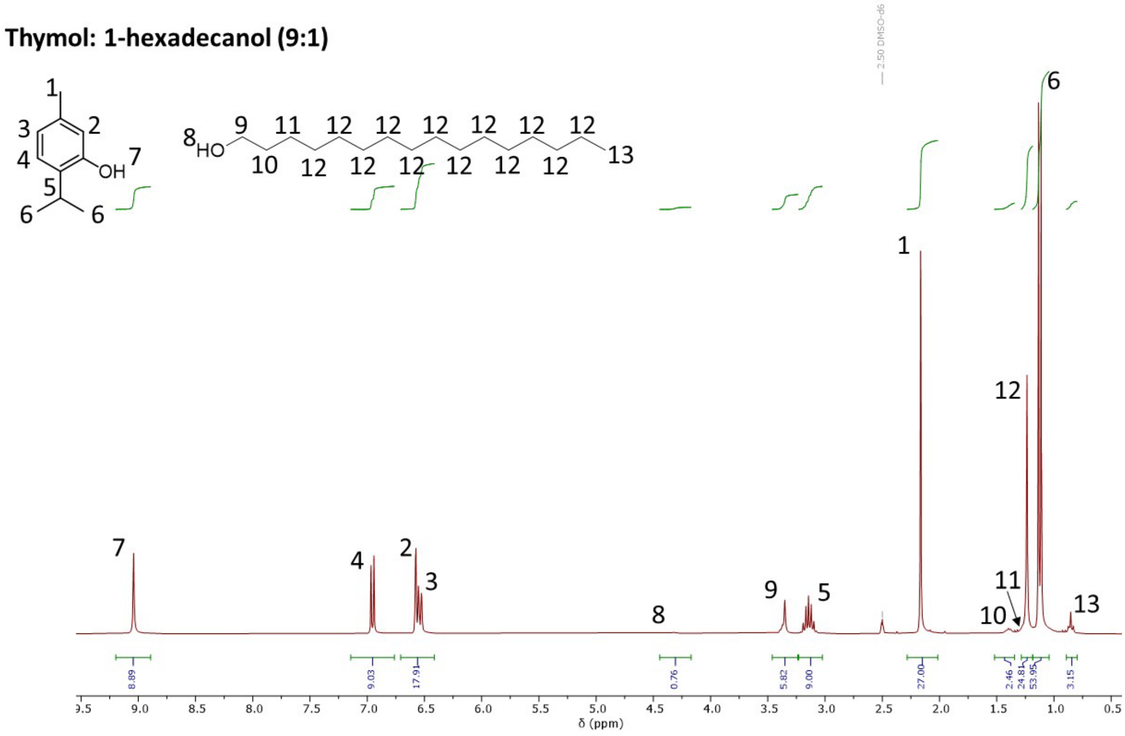

Figure S4. ^{1}H-NMR spectra of DES thymol: 1-hexadecanol (9:1).

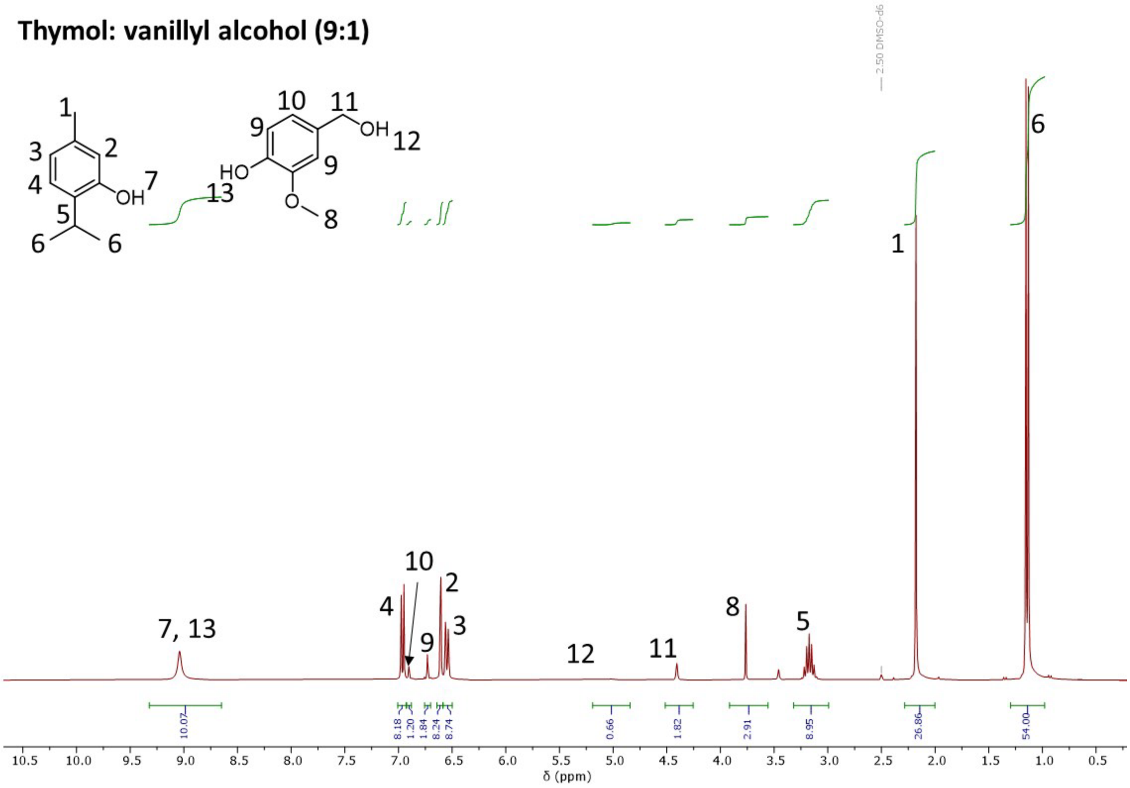

Figure S5. ^{1}H-NMR spectra of DES thymol: vanillyl alcohol (9:1).

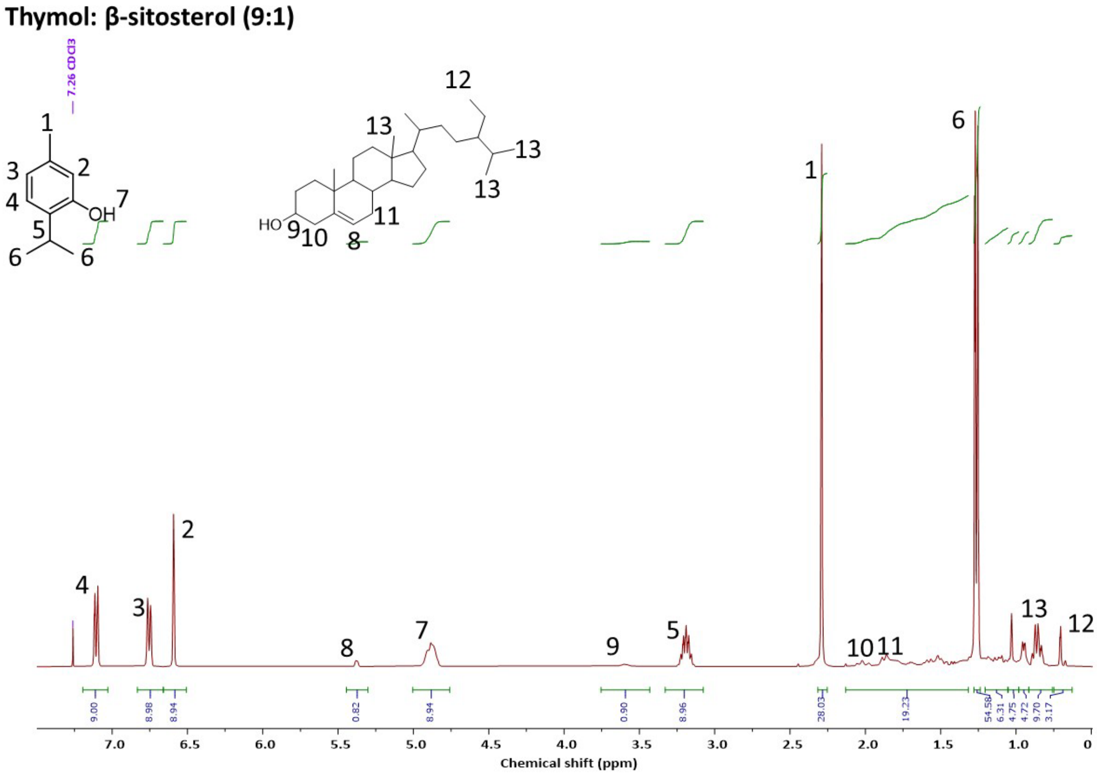

Figure S6. ^{1}H-NMR spectra of DES thymol: β-sitosterol (9:1).

S2. Membrane characterization

50 wt% thymol: 1-hexadecanol (9:1)	50 wt% thymol: β-sitosterol (9:1)	80 wt% thymol: vanillyl alcohol (9:1)
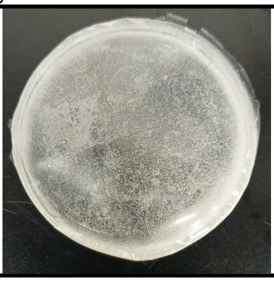	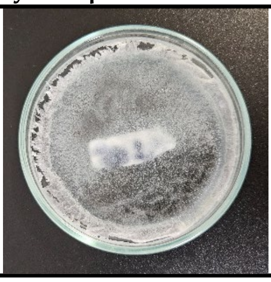	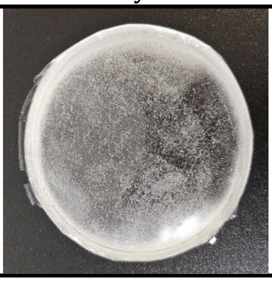

Figure S7: Pictures illustrating unsuccessful attempts at forming ethyl cellulose membranes.

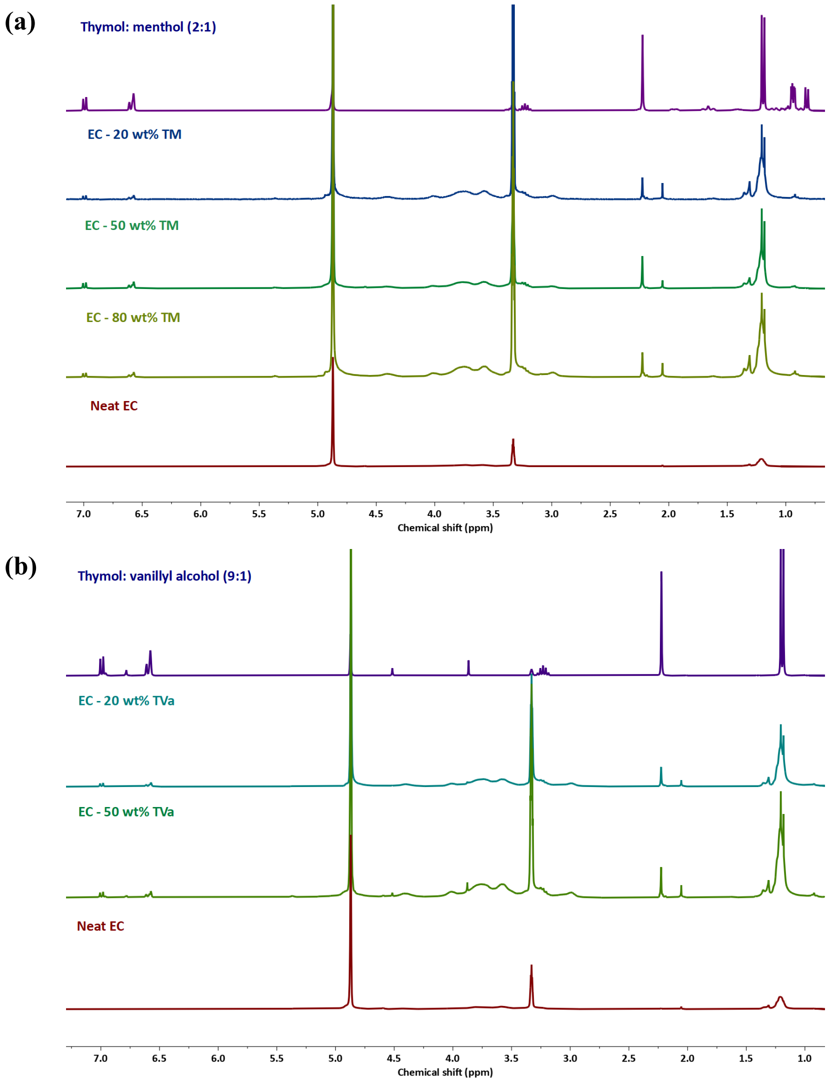

Figure S8. ^{1}H-NMR spectra of neat DES, ethyl cellulose (EC), and the corresponding membranes formed with different amounts of DES **(a)** TM = thymol: menthol (2:1) and **(b)** TVa = thymol: vanillyl alcohol (9:1).

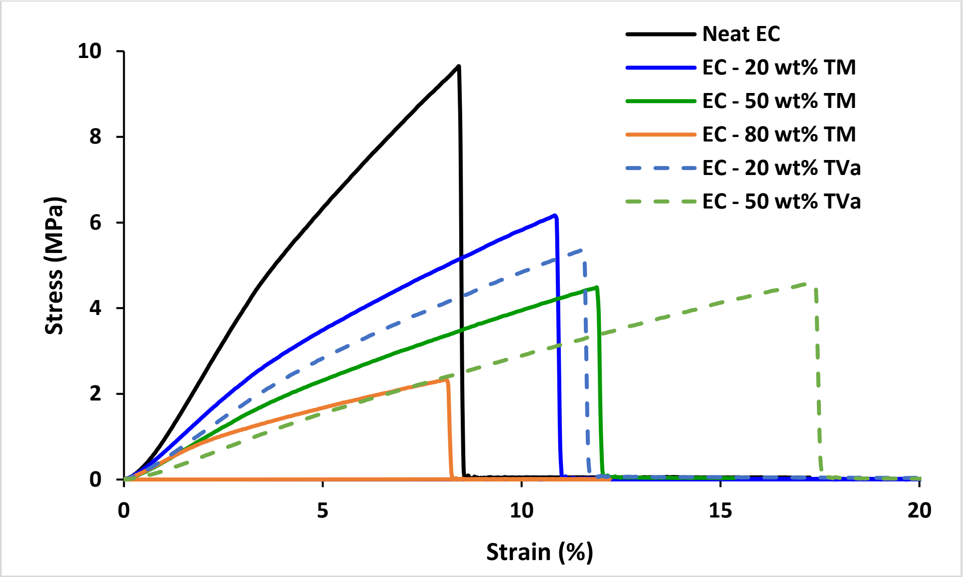

Figure S9. Stress-strain curves of puncture tests depicting the mechanical properties of ethyl cellulose (EC) membranes containing different amounts of DES: TM = thymol: menthol (2:1) and TVa = thymol: vanillyl alcohol (9:1).

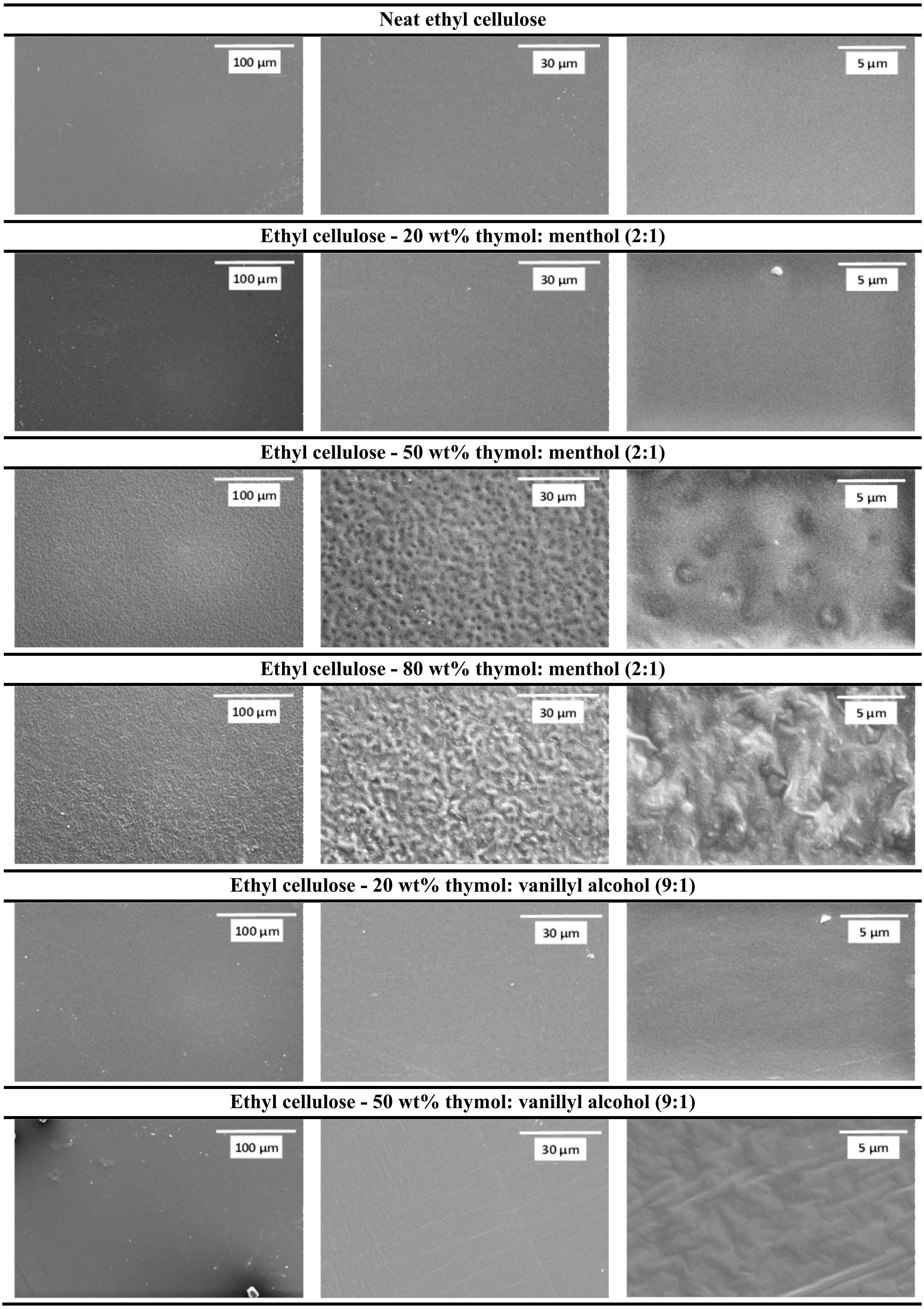

Figure S10. Surface SEM images of ethyl cellulose membranes containing DES at different magnifications.

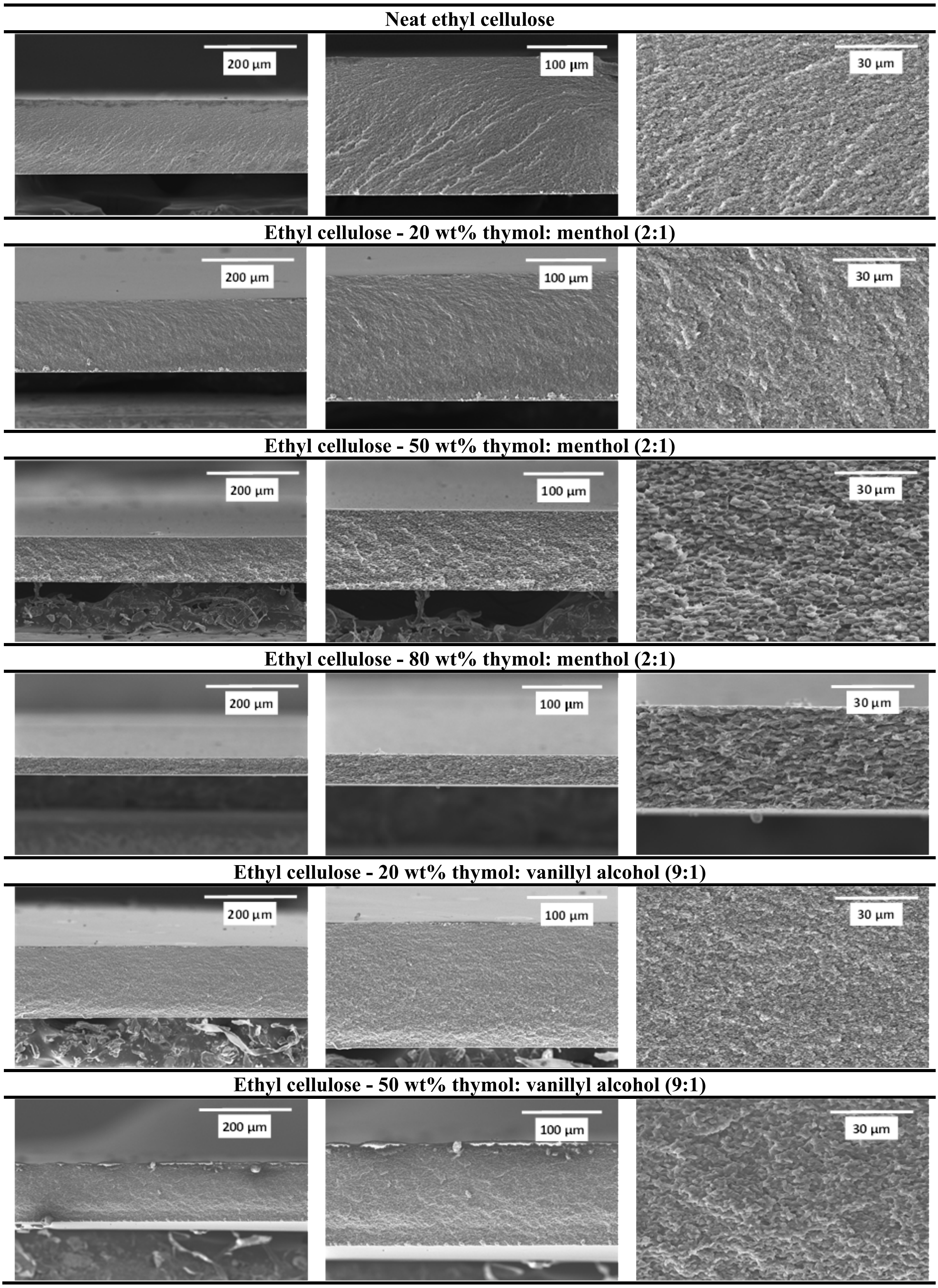

Figure S11. Cross-sectional SEM images of ethyl cellulose membranes containing DES at different magnifications.

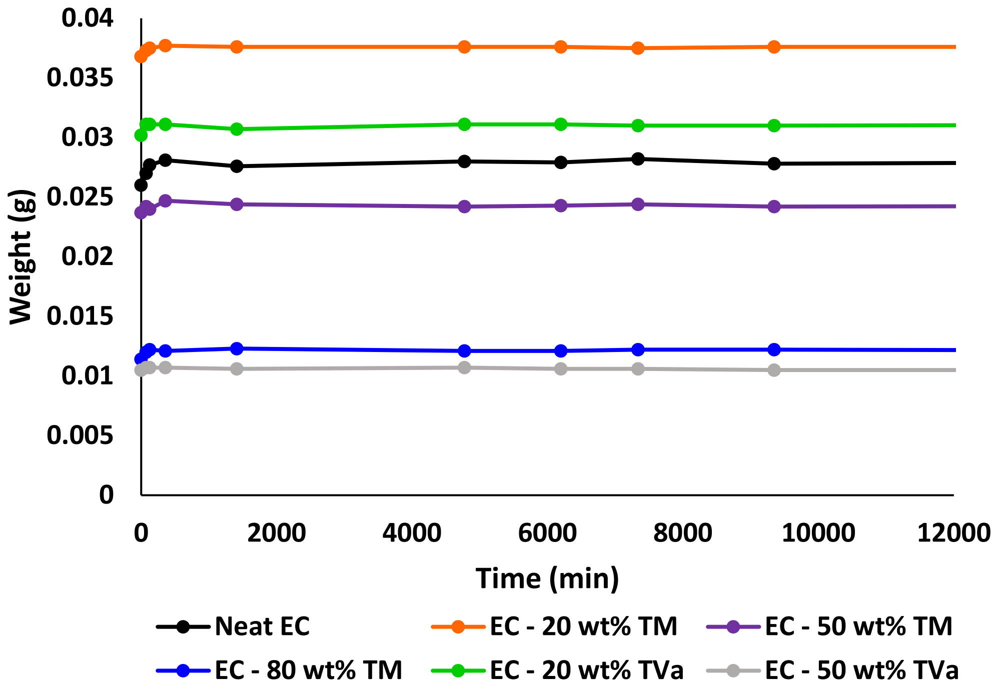

Figure S12. Water up-take measurements for ethyl cellulose (EC) membranes containing different amounts of DES: TM = thymol: menthol (2:1) and TVa = thymol: vanillyl alcohol (9:1).

S3. Eco-scale assessment

Safety evaluation of the reagents [1]

__Thymol__

2.1 Classification of the substance or mixture

Acute toxicity, (Category 4)	H302: Harmful if swallowed.
Skin corrosion, (Sub-category 1B)	H314: Causes severe skin burns and eye damage.
Serious eye damage, (Category 1)	H318: Causes serious eye damage.
Long-term (chronic) aquatic hazard, (Category 2)	H411: Toxic to aquatic life with long lasting effects.

Labelling according Regulation (EC) No 1272/2008

Pictogram

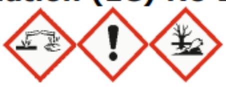

Signal Word	Danger
Hazard Statements	
H302	Harmful if swallowed.
H314	Causes severe skin burns and eye damage.
H411	Toxic to aquatic life with long lasting effects.

__Menthol__

2.1 Classification of the substance or mixture

Classification according to Regulation (EC) No 1272/2008
Skin irritation (Category 2), H315
Eye irritation (Category 2), H319

For the full text of the H-Statements mentioned in this Section, see Section 16.

Labelling according Regulation (EC) No 1272/2008

Pictogram

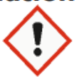

Signal Word	Warning
Hazard statement(s)	
H315	Causes skin irritation.
H319	Causes serious eye irritation.

__Vanillyl alcohol__

2.1 Classification of the substance or mixture

Not a hazardous substance or mixture according to Regulation (EC) No. 1272/2008.

2.2 Label elements

Not a hazardous substance or mixture according to Regulation (EC) No. 1272/2008.

Ethyl cellulose

2.1 Classification of the substance or mixture

Not a hazardous substance or mixture according to Regulation (EC) No 1272/2008.

2.2 Label elements

No hazard pictogram, no signal word, no hazard statement(s), no precautionary statement(s) required

Ethanol

2.1 Classification of the substance or mixture

Flammable liquids, (Category 2)	H225: Highly flammable liquid and vapour.
Eye irritation, (Category 2)	H319: Causes serious eye irritation.

2.2 Label elements

Labelling according Regulation (EC) No 1272/2008

Pictogram

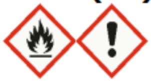

Signal Word Danger

Castor oil

2.1 Classification of the substance or mixture

Not a hazardous substance or mixture according to Regulation (EC) No 1272/2008.

2.2 Label elements

Not a hazardous substance or mixture according to Regulation (EC) No 1272/2008.

Capsaicin

2.1 Classification of the substance or mixture

Classification according to Regulation (EC) No 1272/2008 [EU-GHS/CLP]
Acute toxicity, Oral (Category 3)
Skin irritation (Category 2)
Serious eye damage (Category 1)
Respiratory sensitization (Category 1)
Skin sensitization (Category 1)
Specific target organ toxicity - single exposure (Category 3)

Classification according to EU Directives 67/548/EEC or 1999/45/EC
Toxic if swallowed. Irritating to respiratory system and skin. Risk of serious damage to eyes. May cause sensitization by inhalation and skin contact.

2.2 Label elements

Labelling according Regulation (EC) No 1272/2008 [CLP]

Pictogram

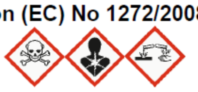

Signal word Danger

Hazard statement(s)

H301	Toxic if swallowed.
H315	Causes skin irritation.
H317	May cause an allergic skin reaction.
H318	Causes serious eye damage.
H334	May cause allergy or asthma symptoms or breathing difficulties if inhaled.
H335	May cause respiratory irritation.

Lipoic acid

2.1 Classification of the substance or mixture

Classification according to Regulation (EC) No 1272/2008
Acute toxicity, Oral (Category 4), H302
Skin irritation (Category 2), H315
Eye irritation (Category 2), H319
Skin sensitization (Category 1), H317
Long-term (chronic) aquatic hazard (Category 2), H411

2.2 Label elements

Labelling according Regulation (EC) No 1272/2008

Pictogram 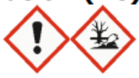

Signal Word	Warning
Hazard statement(s)	
H302	Harmful if swallowed.
H315	Causes skin irritation.
H317	May cause an allergic skin reaction.
H319	Causes serious eye irritation.
H411	Toxic to aquatic life with long lasting effects.

Acetic acid

2.1 Classification of the substance or mixture

Flammable liquids, (Category 3)	H226: Flammable liquid and vapor.
Skin corrosion, (Sub-category 1A)	H314: Causes severe skin burns and eye damage.
Serious eye damage, (Category 1)	H318: Causes serious eye damage.

2.2 Label elements

Labelling according Regulation (EC) No 1272/2008

Pictogram 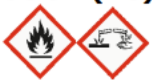

Signal Word	Danger
Hazard Statements	
H226	Flammable liquid and vapor.
H314	Causes severe skin burns and eye damage.

Oleic acid

2.1 Classification of the substance or mixture

Not a hazardous substance or mixture according to Regulation (EC) No. 1272/2008.

2.2 Label elements

No hazard pictogram, no signal word, no hazard statement(s), no precautionary statement(s) required.

Dibutyl sebacate DBS

2.1 Classification of the substance or mixture

Not a hazardous substance or mixture according to Regulation (EC) No 1272/2008.

2.2 Label elements

No hazard pictogram, no signal word, no hazard statement(s), no precautionary statement(s) required

Vitamin D3 Cholecalciferol

2.1 Classification of the substance or mixture

Acute toxicity, (Category 2)	H300: Fatal if swallowed.
Acute toxicity, (Category 2)	H330: Fatal if inhaled.
Acute toxicity, (Category 2)	H310: Fatal in contact with skin.
Specific target organ toxicity - repeated exposure, (Category 1)	H372: Causes damage to organs through prolonged or repeated exposure if swallowed.

2.2 Label elements

Labelling according Regulation (EC) No 1272/2008

Pictogram

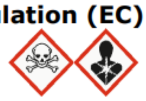

Signal Word	Danger
Hazard Statements	
H300 + H310 + H330	Fatal if swallowed, in contact with skin or if inhaled.
H372	Causes damage to organs through prolonged or repeated exposure if swallowed.

Vitamin E (dl-α-tocopheryl)

2.1 Classification of the substance or mixture

Classification according to Regulation (EC) No 1272/2008

Skin sensitization (Category 1), H317

For the full text of the H-Statements mentioned in this Section, see Section 16.

2.2 Label elements

Labelling according Regulation (EC) No 1272/2008

Pictogram

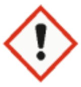

Signal Word	Warning
Hazard statement(s)	
H317	May cause an allergic skin reaction.

Chloroform

2.1 Classification of the substance or mixture

Acute toxicity, (Category 4)	H302: Harmful if swallowed.
Acute toxicity, (Category 3)	H331: Toxic if inhaled.
Skin irritation, (Category 2)	H315: Causes skin irritation.
Eye irritation, (Category 2)	H319: Causes serious eye irritation.
Carcinogenicity, (Category 2)	H351: Suspected of causing cancer.
Reproductive toxicity, (Category 2)	H361d: Suspected of damaging the unborn child.
Specific target organ toxicity - single exposure, (Category 3), Central nervous system	H336: May cause drowsiness or dizziness.
Specific target organ toxicity - repeated exposure, (Category 1), Liver, Kidney	H372: Causes damage to organs through prolonged or repeated exposure if swallowed.

2.2 Label elements

Labelling according Regulation (EC) No 1272/2008

Pictogram 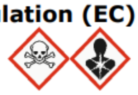

Signal Word: Danger

Hazard Statements

H302	Harmful if swallowed.
H315	Causes skin irritation.
H319	Causes serious eye irritation.
H331	Toxic if inhaled.
H336	May cause drowsiness or dizziness.
H351	Suspected of causing cancer.
H361d	Suspected of damaging the unborn child.
H372	Causes damage to organs (Liver, Kidney) through prolonged or repeated exposure if swallowed.

Table S1. Eco-scale assessment calculations

Parameter Penalty points	Current work		2[2]	3[3]	4[4]	5[5]	6[6]	6[7]
1. Yield (100 – %yield)/2	-	-	-	-	-	-	-	-
2. Price of reaction components (to obtain 10 mmol of end product)	-	-	-	-	-	-	-	-
3. Safety	11	13	30	13	15	5	40	22
4. Technical setup	0	0	0	0	0	0	0	0
5. Temperature/time								
Room temperature, < 1 h 0								
Room temperature, < 24 h 1				1	1	1	1	1
Heating, < 1 h 2					2			
Heating, > 1 h 3	3	3	3	3		3		
Cooling to 0°C 4								
Cooling, < 0°C 5								
6. Workup and purification								
None 0	0	0	0	0		0	0	0
Cooling to room temperature 0								
Adding solvent 0								
Simple filtration 0								
Removal of solvent with bp < 150°C 0								
Crystallization and filtration 1								
Removal of solvent with bp > 150°C 2								
Solid phase extraction 2								
Distillation 3					3			
Sublimation 3								
Liquid-liquid extraction 3								
Classical chromatography 10								
**Eco-scale**	**86**	**84**	**67**	**83**	**79**	**91**	**59**	**77**

References

(1) *Sigma Adrcih (MERCK)*. https://www.sigmaaldrich.com/ (accessed 13 July 2025).
(2) Su, X.; Yang, Z.; Tan, K. B.; Chen, J.; Huang, J.; Li, Q. Preparation and characterization of ethyl cellulose film modified with capsaicin. *Carbohydrate polymers* **2020**, *241*, 116259.
(3) Lin, Y.; Asante, F. O.; Xu, X.; Li, S.; Ding, H.; Xu, L.; Yang, X.; Xia, J.; Li, M. A naturally tailored small molecule for the preparation of ethyl cellulose supramolecular composite film. *Cellulose* **2021**, *28* (1), 289-300.
(4) Mashhadi, H.; Nourabi, A.; Mohammadi, M.; Tabibiazar, M.; Farahani, A. V.; Lorenzo, J. M. Incorporation of myrtle essential oil into hydrolyzed ethyl cellulose films for enhanced antimicrobial packaging applications. *Food Bioscience* **2024**, *62*, 105029.
(5) Hyppölä, R.; Husson, I.; Sundholm, F. Evaluation of physical properties of plasticized ethyl cellulose films cast from ethanol solution Part I. *International journal of pharmaceutics* **1996**, *133* (1-2), 161-170.
(6) Kangarlou, S.; Haririan, I.; Gholipour, Y. Physico-mechanical analysis of free ethyl cellulose films comprised with novel plasticizers of vitamin resources. *International journal of pharmaceutics* **2008**, *356* (1-2), 153-166.
